# Cingulate networks associated with gray matter loss in Parkinson's disease show high expression of cholinergic genes in the healthy brain

**DOI:** 10.1111/ejn.15216

**Published:** 2021-05-04

**Authors:** Arlin Keo, Oleh Dzyubachyk, Jeroen van der Grond, Anne Hafkemeijer, Wilma D.J. van de Berg, Jacobus J. van Hilten, Marcel J.T. Reinders, Ahmed Mahfouz

**Affiliations:** ^1^ Leiden Computational Biology Center Leiden University Medical Center Leiden The Netherlands; ^2^ Delft Bioinformatics Lab Delft University of Technology Delft The Netherlands; ^3^ Department of Radiology Leiden University Medical Center Leiden The Netherlands; ^4^ Department of Methodology and Statistics Institute of Psychology Leiden University Leiden The Netherlands; ^5^ Leiden Institute for Brain and Cognition Leiden University Leiden The Netherlands; ^6^ Department of Anatomy and Neurosciences Amsterdam UMC, location VUmc Amsterdam The Netherlands; ^7^ Department of Neurology Leiden University Medical Center Leiden The Netherlands; ^8^ Department of Human Genetics Leiden University Medical Center Leiden The Netherlands

**Keywords:** Allen Human Brain Atlas, brain imaging, neuroinformatics, spatial transcriptomics, structural covariance networks

## Abstract

Structural covariance networks are able to identify functionally organized brain regions by gray matter volume covariance across a population. We examined the transcriptomic signature of such anatomical networks in the healthy brain using postmortem microarray data from the Allen Human Brain Atlas. A previous study revealed that a posterior cingulate network and anterior cingulate network showed decreased gray matter in brains of Parkinson's disease patients. Therefore, we examined these two anatomical networks to understand the underlying molecular processes that may be involved in Parkinson's disease. Whole brain transcriptomics from the healthy brain revealed upregulation of genes associated with serotonin, GPCR, GABA, glutamate, and RAS‐signaling pathways. Our results also suggest involvement of the cholinergic circuit, in which genes *NPPA*, *SOSTDC1*, and *TYRP1* may play a functional role. Finally, both networks were enriched for genes associated with neuropsychiatric disorders that overlap with Parkinson's disease symptoms. The identified genes and pathways contribute to healthy functions of the posterior and anterior cingulate networks and disruptions to these functions may in turn contribute to the pathological and clinical events observed in Parkinson's disease.

## Graphical abstract

Earlier magnetic resonance imaging (MRI) studies identified brain networks referred to as structural covariance networks (SCNs) for which two were associated with gray matter loss in Parkinson’s disease: the posterior cingulate network and anterior cingulate network. We combined the spatial information from these networks with brain‐wide healthy transcriptomic data from the Allen Human Brain Atlas and found that genes related to the cholinergic system, alcohol and drug withdrawal symptoms, and multiple signaling events, were highly expressed within these brain networks.

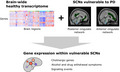

## 1 INTRODUCTION

Parkinson's disease (PD) is a neurodegenerative disorder characterized by the impairment of diverse motor and nonmotor symptoms that get progressively worse over time (Goedert et al., 2012). The decline in clinical performance has been associated with changes in morphological properties of structural and functional neuroimaging networks (Lucas‐Jiménez et al., 2016; de Schipper et al., 2017; Wang et al., 2016). In turn, studies have investigated the relationship between imaging networks and genetic risk factors associated with PD to provide new insights into the pathogenesis of PD (Aarsland et al., 2017; Sampedro et al., 2019; van der Vegt et al., 2009; Winder‐Rhodes et al., 2015). However, less is known about the functions that underlie the spatial organization of brain regions contributing to PD. To identify the molecular mechanisms underlying changes in structural and functional networks in PD, imaging data have been integrated with brain‐wide healthy gene expression from the Allen Human Brain Atlas (AHBA) (Arnatkevic̆iūtė et al., 2019; Hawrylycz et al., 2015). Regional brain atrophy in PD patients was correlated with the expression of genes implicated in trans‐synaptic alpha‐synuclein transfer (Freeze et al., 2018), and a loss of regional connectivity in PD patients was correlated with the regional expression of *MAPT* in the healthy brain (Rittman et al., 2016). These studies showed that combining imaging data in PD and gene expression from the healthy brain can shed light on the molecular mechanisms underlying the morphological differences between PD and controls.

Structural covariance networks (SCNs) identify brain regions that covary in gray matter volume across a population and can reveal functional network organizations (Alexander‐Bloch et al., 2013). SCNs have been shown to be dysregulated in different neurological disorders (Alexander‐Bloch et al., 2013; Coppen et al., 2016; Huang et al., 2017; Liu et al., 2019; Spreng & Turner, 2013), and gray matter variations in SCNs can be explained by transcriptomic similarity and structural connectivity (Romero‐Garcia et al., 2018; Yee et al., 2018). Hafkemeijer et al. (Hafkemeijer et al., 2014) identified nine SCNs based on gray matter variation among healthy middle‐aged to older adults. Gray matter volume in four of these nine networks was negatively associated with age: a subcortical network, sensorimotor network, posterior cingulate networks, and anterior cingulate network. Two of these networks were found to show loss of gray matter volume in PD patients beyond the effects of aging: the posterior cingulate network and anterior cingulate network (de Schipper et al., 2017). Atrophy within these two networks was also associated with cognitive impairment and daytime sleepiness, respectively. Together, these studies revealed how brain networks change in aging and PD, but the molecular mechanisms contributing to the relevant SCNs remain unclear.

Here, we investigated the transcriptomic signatures of the anterior and posterior cingulate networks within the healthy brain. By integrating the nine SCNs with spatial gene expression data from the Allen Human Brain Atlas, we showed that genes highly expressed in the posterior and anterior cingulate networks were associated with multiple neurotransmitter signaling pathways as well as with memory‐related, pain‐related, and neuropsychiatric disorders. In addition, both networks showed high expression of cholinergic marker genes that are known to act as regulators of extracellular signaling. Our results provide new insights into the molecular processes underlying anatomical network function and aids in better understanding the selective progression of PD.

## 2 MATERIALS AND METHODS

### 2.1 Transcriptomic data preprocessing

To understand transcriptomic signatures of nine anatomical networks of the healthy brain, we analyzed gene expression data from the AHBA, a postmortem microarray data set of 3,702 anatomical brain regions from six nonneurological individuals (5 males and 1 female, mean age 42, range 24–57 years) (Hawrylycz et al., 2015). For two out of six donors, samples were available for two hemispheres, while for the remaining four donors there were only samples from the left hemisphere. We analyzed both hemispheres simultaneously whenever this was possible; otherwise, we used data from one hemisphere. Normalized gene expression from the AHBA was downloaded online (http://human.brain‐map.org/). To filter and map probes to genes, the data were concatenated across the six donors. We removed 10,521 probes with missing Entrez IDs, and 6,068 probes with low presence as they were expressed above background in <1% of the samples (PA‐call containing presence/absence flag) (Hawrylycz et al., 2015). The remaining 44,072 probes were mapped to 20,017 genes with unique Entrez IDs using the *collapseRows*‐function in R‐package WGCNA v1.64.1 (Langfelder & Horvath, 2008) as follows: (a) if there is one probe, that one probe is chosen, (b) if there are two probes, the one with maximum variance across all samples is chosen (method=”maxRowVariance”), (c) if there are more than two probes, the probe with the highest connectivity (summed adjacency) is chosen (connectivityBasedCollapsing=TRUE).

For visualization of gene expression in heatmaps, data were Z‐score normalized across all samples for each brain donor separately. Heatmaps were plotted using R‐package ComplexHeatmap v2.0.0 (Gu et al., 2016). Genes were clustered using complete linkage with Euclidean distances. The same color scale for gene expression was used for all heatmaps.

### 2.2 Mapping AHBA samples to SCNs of the healthy brain

We focused on anatomical networks that were previously defined in an MRI study based on whole brain gray matter volume covariation in 370 middle‐aged to older adults between 45 and 85 years (51.9% females) (Hafkemeijer et al., 2014). All subjects in this MRI study did not have a history of psychiatric or neurodegenerative disorders. Written informed consent was obtained from all participants in accordance with the Declaration of Helsinki. The Medical Ethical Committee of the Leiden University Medical Center approved the study. Nine networks were defined and named according to the presence of the main structures: thalamus (Network A), lateral occipital cortex (Network B), posterior cingulate cortex (Network C), anterior cingulate cortex (Network D), temporal pole (Network E), putamen (Network F), and cerebellum (Networks G, H, and I). The same networks were previously investigated for loss of integrity in 159 PD patients from the same age range (36.5% females) (de Schipper et al., 2017). PD patients were recruited from the outpatient clinic for Movement Disorders of the Department of Neurology of Leiden University Medical Center (LUMC) and nearby university and regional hospitals. Written consent was obtained from all participants, and the Medical Ethics Committee of the LUMC approved the study. Samples from each one of the six donors in the AHBA were mapped to regions defined by the nine SCNs in MNI coordinate space. With this mapping, we identified which AHBA samples are located in one of the nine SCNs.

### 2.3 Differential expression analysis

For differential expression analysis we focused on the posterior cingulate network (Network C) and anterior cingulate network (Network D) that were previously associated with gray matter loss in PD (de Schipper et al., 2017). Gene expression in each of the two networks, Network C and Network D, was compared to the other seven networks together (A, B, E, F, G, H, and I). A two‐tailed *t* test was used for each gene and the analysis was done separately for each donor from AHBA. Since the microarray data were log_2_‐transformed, the mean expression difference is interpreted as the log_2_‐transformed fold‐change (FC). The effect sizes for each one of the six donors were combined by meta‐analysis (metafor R‐package 2.0). For the meta‐analysis, a random effects model was applied which assumes that each brain is considered to be from a larger population of brains and therefore takes the within‐brain and between‐brain variance into account. The between‐brain variance (tau^2^) was estimated with the Dersimonian–Delaird model. Variances and confidence intervals were obtained using the *escalc*‐function. The significance of summary effect sizes was assessed through a two‐sided *t* test (H_0_: FC = 0; unequal variances). *P*‐values of the effect sizes were Benjamini–Hochberg (BH) corrected for all 20,017 genes. Genes were differentially expressed within the posterior cingulate network or the anterior cingulate network compared to the other networks combined when the absolute FC > 1 and the BH‐corrected *p*‐value < .05. To asses the reproducibility of the differentially expressed genes, we calculated the differential stability of all 20,017 genes in our dataset. This value was calculated as the average Pearson's correlation between all 15 possible pairs of six donors from the AHBA. The individual correlations for each pair of donors were calculated across samples that were shared between two donors.

### 2.4 Functional enrichment analysis

Pathway analysis was done with the ReactomePA R‐package version 1.28 using the function *enrichPathway* searching for human pathways. All 20,017 genes in the AHBA dataset were set as background genes. Pathways with a minimum size of 10 genes and BH‐corrected *p* < .05 were considered significant. An additional functional enrichment test for GO‐terms was done with clusterProfiler R‐package version 3.18.1. The same background genes were used as before and GO‐terms with BH‐corrected *p* < .05 were considered significant.

### 2.5 Cell‐type marker enrichment

Gene markers for 28 cell‐types were downloaded from the NeuroExpresso database (http://neuroexpresso.org/) using markers from all brain regions. These have been identified in a cross‐laboratory dataset of cell‐type specific transcriptomes from the mouse brain (Mancarci et al., 2017). To assess their expression, Entrez IDs of the mouse cell‐type specific markers were converted to human homologs (homologene R‐package version 1.4) and filtered for genes present in the AHBA dataset (Table S1). Two markers with different mouse gene IDs (14,972, *H2‐K1*, microglial, and 15,006, *H2‐Q1* serotonergic) were converted to the same human gene ID (3,105, *HLA‐A*) and therefore removed before analysis. For cell‐type enrichment, we assessed which cell‐type markers were overrepresented among the differentially expressed genes. For 17 cell‐types that had at least six markers (astrocyte, Bergmann, cerebellar granule, dentate granule, ependymal, GabaReln, hypocretinergic, microglia, activated microglia, deactivated microglia, noradrenergic, oligo, purkinje, serotonergic, spinal cord cholinergic, spiny, and thalamus cholinergic), we assessed the significance with the hypergeometric test and *p*‐values were corrected for all 17 cell types (BH‐corrected *p* < .05).

We performed an additional functional enrichment test with expression weighted cell‐type enrichment (EWCE) analysis (Skene & Grant, 2016) that makes use of single‐cell transcriptome data to estimate the probability of a gene list being associated with a cell‐type. For this purpose, we processed cell‐type data from the NeuroExpresso database and selected gene markers for 28 cell‐types that were proposed by NeuroExpresso. BH‐corrected *p*‐values < .05 were considered significant.

### 2.6 Enrichment of disease‐associated genes

Differentially expressed genes were also assessed for the overrepresentation of disease‐associated genes from DisGeNET (Piñero et al., 2017). A table of 628,685 gene‐disease associations was obtained from DisGeNET version 6.0 (July 2019) from http://www.disgenet.org/ website. A hypergeometric test was used to assess the significance of overlapping genes (*p* < .05), and *p*‐values were BH‐corrected for 24,166 diseases. The odds ratio (OR) for cell‐type and disease enrichment was calculated using the DescTools R‐package.

## 3 RESULTS

### 3.1 Transcriptomics of the posterior and anterior cingulate networks

**FIGURE 1:**
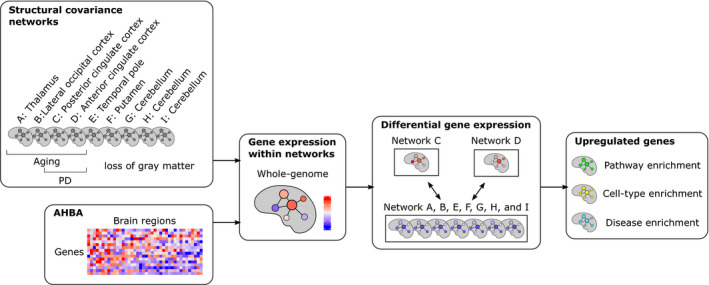
Study overview. Transcriptomic data from the Allen Human Brain Atlas (AHBA) were mapped to nine anatomical networks that have been defined based on healthy subjects. Network C (posterior cingulate network) and Network D (anterior cingulate network) have been associated with gray matter loss in Parkinson**'**s disease (PD), while the seven remaining networks were not related to PD. We compared gene expression in Network C and Network D to gene expression in Networks A, B, E, F, G, H, and I together. Upregulated genes were assessed for the overrepresentation of pathway‐specific genes, cell‐type marker genes, and disease‐associated genes

**FIGURE 2:**
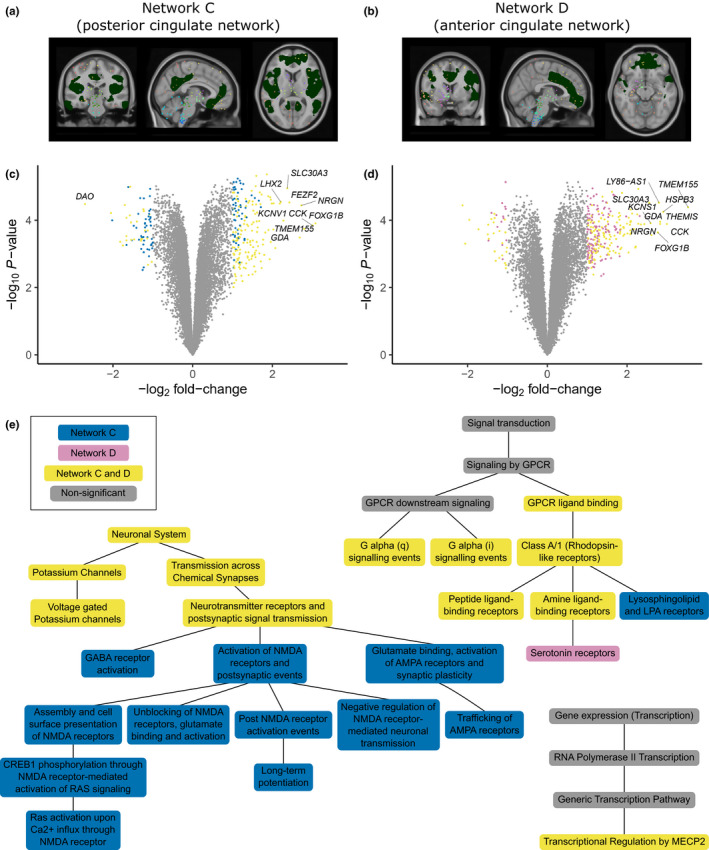
Differential gene expression analysis of structural covariance networks associated with Parkinson**'**s disease. (a, b) Brain regions of interest (green) defined by structural covariance networks (SCNs), Network C (posterior cingulate network), and Network D (anterior cingulate network) that were identified in a previous study (Hafkemeijer et al., 2014). Colored points correspond to the spatial location of Allen Human Brain Atlas (AHBA) samples where colors represent different anatomical structures. AHBA samples were mapped to SCNs based on their position inside or outside the regions of interest. Genes were analyzed for differential expression in (c) Network C and (d) Network D compared to seven other SCNs. Effect sizes were summarized across the six healthy donors from the AHBA with meta‐analysis. For all genes (points) the log_2_ fold‐change (FC; *x*‐axis) and −log_10_ of nominal *p*‐values (*y*‐axis) are shown. Significant differentially expressed genes (*t* test, BH‐corrected *p* < .05, and |FC| > 1) are unique for each network (blue and purple points) or significant in both networks (yellow points). The top 10 genes with the highest absolute FC are labeled for each network and highly overlap between both networks. (e) Pathway analysis of differentially upregulated genes in Network C and Network D shows similar enriched pathways (yellow) that are hierarchically organized in the Reactome database. Network C showed more specific associations with pathways involved in neurotransmitter receptors and postsynaptic signal transmission (blue). Network D was more specifically associated with serotonin receptors (purple). See Table S6 for gene counts and BH‐corrected *p*‐values

We analyzed the transcriptomes of healthy subjects across nine anatomical networks defined by structural covariance of gray matter volume among healthy middle‐aged to older adults (Hafkemeijer et al., 2014). For this we used the AHBA microarray dataset of spatial gene expression in postmortem brains of six nonneurological donors and samples were mapped to each one of the nine Networks A‐I (Table 1) based on their spatial location (Figure 1). We focused on the posterior cingulate network (Network C) and the anterior cingulate network (Network D) that showed loss of gray matter in PD patients (Figure 2a,b) (de Schipper et al., 2017) and characterized their transcriptional signatures by comparing them to the remaining seven networks together.

**TABLE 1:** Number of samples from the Allen Human Brain Atlas (AHBA) that fall within networks A‐I

Donors	Network								
	A	B	C	D	E	F	G	H	I
Donor 9,861	72	67	157	47	74	90	26	39	83
Donor 10,021	79	46	121	65	49	84	25	55	91
Donor 12,876	37	24	57	28	42	45	6	17	25
Donor 14,380	38	33	52	30	45	61	7	27	53
Donor 15,496	34	24	41	21	39	55	13	24	69
Donor 15,697	49	20	38	33	47	64	29	37	49
Total	309	214	466	224	296	399	106	199	370

Note

A: Thalamus; B: Lateral occipital cortex, C: Posterior cingulate cortex, D: Anterior cingulate cortex, E: Temporal pole; F: Putamen; G, H, I: Cerebellum.

Whole genome differential expression analysis showed a large overlap of genes that were differentially expressed in the same direction in the two networks. We found that 73 genes in Network C and 39 genes in Network D were downregulated, of which 25 genes overlapped between both networks (Figure 2c,d and Tables S2 and S3). Furthermore, 200 genes in Network C and 269 genes in Network D were upregulated, for which 144 genes overlapped (Tables S4 and S5). To find out whether our significant genes have reproducible expression across the six donors, we assessed the differential stability, which is the average Pearson's correlation between all 15 possible pairs of the six donors, an approach that has previously been applied to the same dataset (Hawrylycz et al., 2015). Most differentially expressed genes (>92%) were among the top decile of all 20,017 genes corresponding to a differential stability value >0.73 (Figure S1). Among the differentially expressed genes in the posterior and anterior cingulate networks, no PD‐implicated genes were found that arouse from familial and genome‐wide association studies (Bonifati, 2014; Chang et al., 2017; Nalls et al., 2014).

For functional interpretation of the differentially upregulated genes, we further assessed the enrichment of genes associated with biological pathways in the Reactome Pathway Database (see Methods, Table S6). As both Networks C and D shared many differentially expressed genes, they also shared similar pathways: transcriptional regulation by *MECP2*, GPCR (G protein‐coupled receptor) signaling, voltage gated potassium channels, and neurotransmitter receptor and postsynaptic signal transmission (Figure 2e). For better interpretation, we assessed the hierarchical relationships between enriched pathways based on the ontology of the Reactome Pathway Database. Pathways that describe more general biological functions are found at the top of the hierarchy (closer to the root) and were enriched for both Networks C and D. Pathways that describe more specific biological functions are lower in the hierarchy and were enriched for either Network C or Network D. Network C was additionally related to more specific pathways such as lysosphingolipid and LPA receptors, GABA receptor activation, RAS‐signaling mediated by NMDA receptors, glutamate binding, activation of AMPA receptors and synaptic plasticity, and long‐term potentiation. Network D was additionally associated with serotonin receptors. To verify our results, we performed another functional analysis and assessed the enrichment of Gene Ontology (GO) terms. Again, Network C and Network D shared similar functional terms, for example, potassium ion transport, GPCR signaling pathway, and regulation of neurotransmitter receptor activity. Overall, we found GO terms that were similar to the pathways identified with Reactome (Table S7 and S8).

### 3.2 Cholinergic cell markers are highly expressed within cingulate networks

**FIGURE 3:**
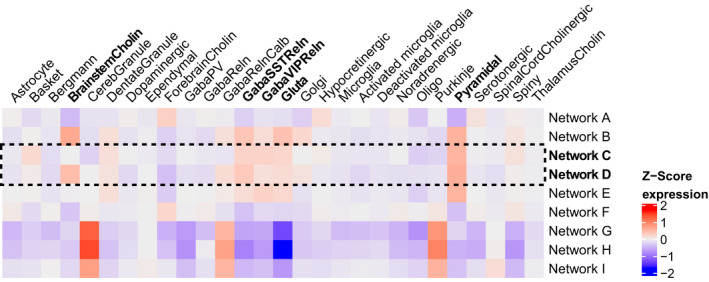
Expression of cell‐types in anatomical networks. Gene expression was Z‐scored and averaged across cell‐type specific markers, across samples within anatomical networks, and across the six donors in the AHBA. Networks G, H, and I are cerebellar networks and thus showed distinct expression patterns. Network C (posterior cingulate network) and Network D (anterior cingulate network) showed high expression of marker genes for brainstem cholinergic cells, GabaSSTReln, GabaVIPReln, glutamatergic cells, and pyramidal cells. Gene expression heatmaps for each donor are shown in Figure S2

The composition of specific cell‐types can shape the transcriptomic features of anatomical networks. Therefore, we analyzed whether genes differentially expressed in the posterior and anterior cingulate networks were enriched for cell‐type specific marker genes from the NeuroExpresso database (Mancarci et al., 2017). To assess the expression of cell‐types, we averaged the expression of marker genes associated with a cell‐type. Both Network C and Network D showed high expression of marker genes for brainstem cholinergic cells, GabaSSTReln, GabaVIPReln, glutamatergic, and pyramidal cells (Figure 3 and Figure S2).

**FIGURE 4:**
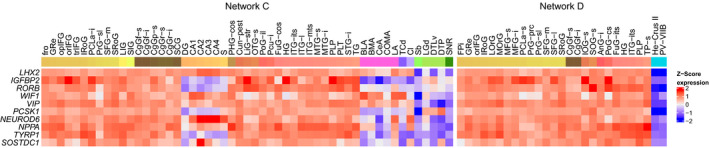
Expression of differentially upregulated cell‐type marker genes in Network C (posterior cingulate network) and Network D (anterior cingulate network). Heatmaps of differentially expressed marker genes (rows) are shown for one of the six donors in the Allen Human Brain Atlas (donor 10,021). Samples from different anatomical substructures within the networks are color annotated (columns). Expression was averaged across samples from an anatomical substructure with the same acronym ignoring left and right hemisphere annotations. See Figure S3 for heatmaps of all six donors from the AHBA and Table S9 for full names of the region‐specific acronyms

Among the differentially upregulated genes in Network C and Network D, we found 10 marker genes representing six cell‐types: astrocyte, Bergmann, GabaVIPReln, hypocretinergic, pyramidal, and thalamus cholinergic (Table 2). Markers that were significantly upregulated in Network C were also significantly upregulated in Network D. In both networks, the 10 markers were highly expressed in cortical regions, including the cingulate gyrus and lowly expressed in limbic regions (Figure 4 and Figure S3).

**TABLE 2:** Differentially upregulated cell‐type marker genes in Network C (posterior cingulate network) and Network D (anterior cingulate network)

Gene	Marker	Network C		Network D	
		FC	BH	Estimate	BH
*LHX2*	Astrocyte	**2.21**	**3.92E‐03**	**2.00**	**6.46E‐03**
*IGFBP2*	Astrocyte	0.69	5.80E‐02	**1.18**	**1.78E‐02**
*RORB*	Astrocyte	0.82	**3.09E‐02**	**1.19**	**1.39E‐02**
*WIF1*	Bergmann	**1.02**	**8.74E‐03**	**1.03**	**7.95E‐03**
*VIP*	GabaVIPReln	**1.67**	**4.23E‐03**	**1.85**	**6.89E‐03**
*PCSK1*	Hypocretinergic	**1.15**	**1.25E‐02**	**1.57**	**1.06E‐02**
*NEUROD6*	Pyramidal	**1.90**	**4.78E‐03**	**1.92**	**6.76E‐03**
*NPPA*	ThalamusCholin	**1.64**	**6.98E‐03**	**2.09**	**6.39E‐03**
*TYRP1*	ThalamusCholin	0.81	**2.41E‐02**	**1.43**	**9.82E‐03**
*SOSTDC1*	ThalamusCholin	0.83	**1.21E‐02**	**1.14**	**6.39E‐03**

Note

Fold‐change (FC) and Benjamini–Hochberg (BH) corrected *p*‐value are shown for cell‐type marker genes that were differentially expressed in the two networks compared to the remaining networks. FC >1 and BH <0.05 are highlighted in bold text.

**FIGURE 5:**
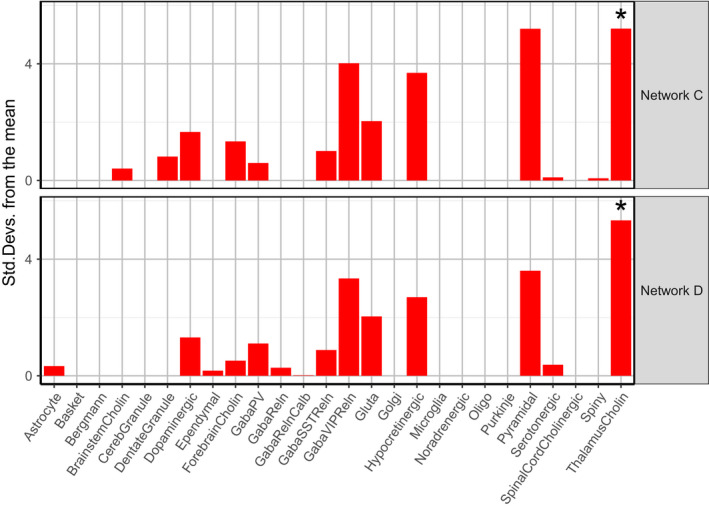
Expression weighted cell‐type enrichment (EWCE) analysis of cell‐types for upregulated genes in Network C (posterior cingulate network) and Network D (anterior cingulate network). Cell‐type expression and selection of cell‐type informative genes (markers) are based on the NeuroExpresso database

Cell‐type enrichment analysis revealed that only markers for thalamus cholinergic cells were significantly overrepresented among genes that were upregulated in Network D (OR = 17.12 and *p* = 2.01e‐02). The responsible markers *NPPA*, *SOSTDC1*, and *TYRP1* showed high expression within Network D, as well as in most parts of Network C (Figure 4). An additional enrichment analysis that makes use of single cell transcriptome data (EWCE) revealed that genes upregulated in both Networks C and D were significantly enriched for thalamus cholinergic cells (Figure 5). Interestingly, while other thalamus cholinergic marker genes showed high expression in limbic samples and low expression in cortical samples within both networks, *NPPA*, *SOSTDC1*, and *TYRP1* showed opposite expression patterns with low expression in limbic samples, including the thalamus, and high expression in cortical samples (Figure S4).

### 3.3 Cingulate networks are enriched for genes associated with disorders relevant to PD

**FIGURE 6:**
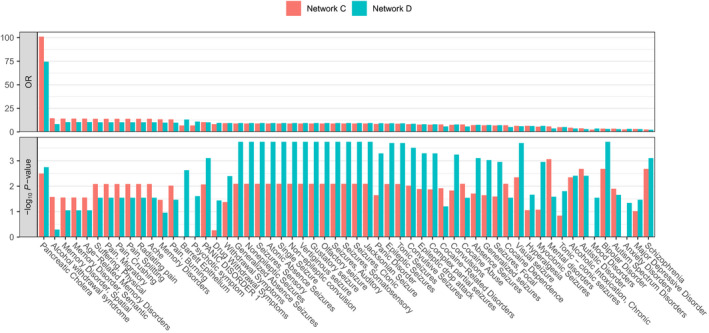
Disease associations of Network C (posterior cingulate network) and Network D (anterior cingulate network). Differentially upregulated genes in each network were assessed for the enrichment of disease‐associated genes from DisGeNET (hypergeometric test, BH‐corrected *p* < .05). Top plot shows odds ratios (ORs) for the number of overlapping genes, and bottom plot shows the significance of overlap indicated with –log_10_ *p*‐values (*y*‐axis). Disorders (columns) are sorted based on highest ORs in either one of the networks

Dysregulation of functional networks may result in a broader spectrum of disorders than PD. Therefore, we assessed which disease‐associated genes from DisGeNET were overrepresented among the differentially upregulated genes in Network C as well as Network D. Since both networks shared many upregulated genes, similar disease associations were also found. We found that genes upregulated in both networks were significantly associated with epileptic and nonepileptic seizures, many mental disorders (bipolar, panic, autistic, [age‐related] memory, mood, major depressive, and anxiety disorder), pain, and schizophrenia (Figure 6). Network C, the posterior cingulate network, was more related to memory and pain‐related disorders, while Network D, the anterior cingulate network, was more related to mental and neuropsychiatric disorders. Furthermore, we found that differentially expressed genes were associated with disorders related to alcohol and drug abuse. These included withdrawal symptoms, drug withdrawal symptoms, alcohol withdrawal syndrome, cocaine dependence, cocaine abuse, and cocaine‐related disorders. In summary, we found associations with disorders that relate to defects in brain functions that are relevant to PD.

## 4 DISCUSSION

We examined transcriptomic signatures of the healthy brain in brain regions defined by SCNs that were identified in an earlier imaging analysis study (Hafkemeijer et al., 2014). In particular, we focused on molecular mechanism underlying two SCNs that were previously associated with decreased gray matter in PD patients (de Schipper et al., 2017) and were named the posterior cingulate network (Network C) and anterior cingulate network (Network D) as they mostly covered these anatomical areas. Pathway analysis revealed genes related to GPCR signaling, transcriptional regulation by *MECP2*, and neurotransmitter receptors and postsynaptic signal transmission. We found that genes that were upregulated in the posterior cingulate gyrus and anterior cingulate gyrus were also enriched for thalamus cholinergic marker genes. Moreover, our results showed that both SCNs are associated with multiple neurotransmitter signaling pathways, for example, serotonin, GPCR, GABA, glutamate, and RAS.

Genes that were highly expressed in the anterior cingulate network were significantly enriched for thalamus cholinergic markers, specifically: *NPPA*, *SOSTDC1*, and *TYRP1*. These marker genes, together with other markers of this cell‐type, were previously defined based on their expression in cholinergic cells from the mouse thalamus, more specifically the hubenula (Mancarci et al., 2017). According to the AHBA ontology, the hubenula is not part of the thalamus. In this study, most thalamus cholinergic marker genes indeed showed high expression in human thalamic regions. However, *NPPA*, *SOSTDC1*, and *TYRP1* unexpectedly showed opposite expression patterns with mainly high expression in cortical regions and low expression in limbic regions, including the thalamus. Cholinergic circuits are key in cognitive functions, and cholinergic denervation of the cortex and thalamus in PD patients may contribute to the transition from PD to PD with dementia (Ballinger et al., 2016). We found that glutamatergic and GABAergic marker genes were also highly expressed within the posterior and anterior cingulate networks, although statistical significance could not be assessed due to the small number of marker genes for these cell‐types. Interestingly, acetylcholine release by cholinergic neurons affects glutamatergic and GABAergic signaling by altering the synaptic excitability (Buendia et al., 2019; Granger et al., 2015). Moreover, it is thought that dysfunction of cholinergic circuits contributes to cognitive decline associated with neurodegenerative diseases (Ballinger et al., 2016).

Cholinergic marker genes *NPPA*, *SOSTDC1*, and *TYRP1* were highly expressed in the posterior cingulate network and anterior cingulate network of the healthy brain compared to the other seven SCNs. While the functions of these genes likely involve cholinergic signaling, several studies suggest that they also function as extracellular regulators of multiple other signaling pathways, including cAMP, Wnt, and β‐catenin signaling (Bansho et al., 2017; Brenner et al., 1990; De Vito, 2014; Hirobe, 2011; Kutchko & Siltberg‐Liberles, 2013; Millan et al., 2019).

*NPPA* (natriuretic peptide precursor A) and other natriuretic peptides are thought to be involved in a wide range of functions, including neurovascular functions, blood‐brain barrier, brain homeostasis, neuroprotection, and synaptic transmission by regulating the release and re‐uptake of neurotransmitters such as noradrenalin, dopamine, and glycine (Mahinrad et al., 2016). Impaired function of natriuretic peptides in brains of AD patients could accelerate neurodegeneration and may impair structural integrity of the brain leading to a higher risk of cognitive decline (Mahinrad et al., 2018). Our results suggest that *NPPA* might similarly be involved in PD pathogenesis given its high expression within the anterior and posterior cingulate networks.

*SOSTDC1* (sclerostin domain‐containing 1) is known as a negative regulator of bone morphogenetic protein (BMP) and Wnt‐signaling, but recent studies also show that *SOSTDC1* regulates natural killer cell maturation and cytotoxicity (Millan et al., 2019). An increased number of natural killer cells have been found in PD, but the actual relevance with PD risk is still unclear (Jiang et al., 2017). The BMP signaling pathway promotes the development of midbrain dopaminergic neurons (Jovanovic et al., 2018), in which *SOSTDC1* may play a role. Furthermore, *SOSTDC1* was upregulated in the striatum of Parkinsonian rats that were treated by subthalamic nucleus high‐frequency stimulation and is therefore suggested to have neuroprotective effects (Lortet et al., 2013).

*TYRP1* (tyrosinase‐related protein 1) produces melanocytes‐specific proteins involved in the biosynthesis of melanin in brain, skin, and eyes (Lu et al., 2011; Wang & Hebert, 2006). Melanoma and PD share genes involved in the synthesis of melanin and dopamine, including *SNCA* which encodes the α‐synuclein protein found in Lewy bodies (Pan et al., 2012). Furthermore, neuromelanin is produced almost exclusively in human catecholaminergic neurons and is responsible for the pigmentation of dopaminergic neurons of the substantia nigra and noradrenergic neurons of the locus cereleus (Pavan & Dalpiaz, 2017). It is considered to be protective due to its ability to chelate metals, especially iron for which levels increases with age (Pavan & Dalpiaz, 2017).

The posterior and anterior cingulate networks shared similar highly expressed genes and were likewise associated with similar diseases. Based on our analysis of transcriptomic signatures in the healthy brain, we found that the posterior cingulate network showed stronger associations with memory and pain‐related disorders compared to the anterior cingulate networks which showed stronger associations with mental and neuropsychiatric disorders. As part of the default mode network, both the posterior and anterior cingulate cortex have been shown to be dysregulated in neuropsychiatric disorders (Broyd et al., 2009; Öngür et al., 2010). We also found that both networks were associated with alcohol and drug withdrawal symptoms and more specifically cocaine‐related disorders. Cocaine abuse has been ambiguously related to PD, for example, cocaine binds to dopamine transport proteins, and cocaine users show excess iron accumulation in the brain. However, there has been no direct association between cocaine usage and an increasing risk to develop PD (Ball et al., 2019). Furthermore, alcohol use disorder has been associated with neurodegenerative diseases, including Alzheimer's disease and PD, as chronic alcohol intake can induce oxidative stress and trigger the neuroimmune response and excitotoxicity (Kamal et al., 2020).

Although we are interested in brain regions that are vulnerable to PD, our study is limited to transcriptomic data from the healthy brain. In this study, the regions of interest are defined by brain networks based on SCNs and one such a network can comprise of multiple distant and disconnected regions. Therefore, a region of interest in this study cannot be compared with the typical anatomical structures that have been analyzed in previous PD transcriptomic studies. In addition, validation with PD brains is challenging due to the scarcity of spatial transcriptomic data of PD brains. There are few studies that analyzed multiple brain regions in PD, but they only cover few brain regions of interest. To map transcriptomic samples to brain regions defined by SCNs, a high spatial resolution is needed for the transcriptomic data, which is currently not available for PD. Therefore, it will be interesting for future studies to profile the transcriptomes of PD brain regions at a higher spatial resolution.

In transcriptional maps, such as the AHBA, samples are strongly spatially autocorrelated meaning that nearby brain regions share more similar expression patterns than distant brain regions (Fulcher et al., 2020). This may cause a bias in enrichment analyses towards gene sets that are higher co‐expressed in the brain and thus describe more general brain‐related functions. While there are interesting methods to correct for this spatial bias, they are still being developed. In addition, we believe that our results are not affected by spatial autocorrelation, as our regions of interests, two SCNs, consist of separate distant brain regions that span parts of multiple anatomical brain regions.

In summary, our results highlight molecular mechanisms that underlie two specific SCNs in the healthy brain: the posterior cingulate network and anterior cingulate network. Both SCNs represent anatomical networks that function normally in healthy brains, but their activity is reduced in aging and PD (Hafkemeijer et al., 2014; de Schipper et al., 2017). Our findings suggest that genes involved in multiple signaling pathways, such as serotonin, GPCR, GABA, glutamate, and RAS, contribute to healthy functions of the posterior and anterior cingulate networks. While these observations apply to the healthy brain, they provide insight into the structures that are vulnerable in PD. Further research will be needed to better understand the transcriptomics of brain networks and how they are involved in PD.

## CONFLICT OF INTEREST

The authors declare no competing interests.

## AUTHOR CONTRIBUTIONS

AK, JJH, MR, and AM designed the study. Imaging data were provided by JG and AH and processed by OD. AK performed the data analysis. AK, WDJB, JJH, MR, and AM interpreted the data and wrote the manuscript with input from all authors. AM and MR supervised the overall project. The final manuscript was read and approved by all authors.

### PEER REVIEW

The peer review history for this article is available at https://publons.com/publon/10.1111/ejn.15216.

## Supporting information

Supplementary Material

## Data Availability

Transcriptomic data from the AHBA are publicly available online (http://human.brain‐map.org/). Imaging data are available upon request. Scripts to run all analyses can be found online at https://github.com/arlinkeo/pd_scn and were run in R version 4.
