# Supplementary material for: Cingulate networks associated with gray matter loss in Parkinson's disease show high expression of cholinergic genes in the healthy brain
Source: Eur J Neurosci. 2021 May 4;53(11):3727–39. doi: 10.1111/ejn.15216 (PMC8251922; doi:10.1111/ejn.15216)
Supplement: Supplementary file 1 — Supplementary Material [file EJN-53-3727-s001.pdf]

Supplementary Materials

Cingulate networks associated with gray matter loss in Parkinson's disease show high expression of cholinergic genes in the healthy brain

Arlin Keo[1,2], Oleh Dzyubachyk[3], Jeroen van der Grond[3], Anne Hafkemeijer[3,4,5], Wilma D.J. van de Berg[6], Jacobus J. van Hilten[7], Marcel J. T. Reinders[1,2], Ahmed Mahfouz[1,2,8*].

1. Leiden Computational Biology Center, Leiden University Medical Center, Leiden, The Netherlands.
2. Delft Bioinformatics Lab, Delft University of Technology, Delft, The Netherlands.
3. Department of Radiology, Leiden University Medical Center, Leiden, The Netherlands.
4. Department of Methodology and Statistics, Institute of Psychology, Leiden University, Leiden, the Netherlands.
5. Leiden Institute for Brain and Cognition, Leiden University, Leiden, the Netherlands.
6. Department of Anatomy and Neurosciences, Amsterdam Neuroscience, Amsterdam UMC, location VUmc, Amsterdam, The Netherlands.
7. Department of Neurology, Leiden University Medical Center, Leiden, The Netherlands.
8. Department of Human Genetics, Leiden University Medical Center, Leiden, The Netherlands.

*a.mahfouz@lumc.nl

Supplementary Tables

Supplementary Table 1 Number of cell-type markers after conversion to human homologs and filtering for genes present in AHBA data. Cell-type marker genes from NeuroExpresso were based on mouse gene expression data.

Cell-type	Mouse	Human	Cell-type	Mouse	Human
Astrocyte	97	95	Glutamatergic	1	1
Basket	1	1	Golgi	4	4
Bergmann	32	30	Hypocretinergic	10	10
Brainstem cholinergic	1	1	Microglia	122	120
Cerebellar granule	9	9	Activated microglia	88	85
Dentate granule	7	7	Deactivated microglia	107	103
Dopaminergic	1	1	Noradrenergic	8	8
Ependymal	45	42	Oligo	23	23
Forebrain cholinergic	5	5	Purkinje	27	27
GabaPV	3	3	Pyramidal	1	1
GabaReln	6	6	Serotonergic	7	7
GabaRelnCalb	1	1	SpinalCord cholinergic	7	6
GabaSSTReln	5	4	Spiny	10	10
GabaVIPReln	5	5	Thalamus cholinergic	16	16

Supplementary Table 2 Differentially downregulated genes in Network C (posterior cingulate network).

Gene name	Entrez ID	Fold-change	lower95	upper95	P-value	BH	Differential stability
ALOX5	240	-1.07	-1.22	-0.91	1.16E-05	3.92E-03	0.82
ANK1	286	-1.06	-1.26	-0.85	4.76E-05	3.92E-03	0.88
CDH7	1005	-1.09	-1.31	-0.87	5.28E-05	3.92E-03	0.86
CHRNA3	1136	-1.08	-1.31	-0.85	6.69E-05	3.92E-03	0.84
DAO	1610	-2.71	-3.20	-2.21	3.29E-05	3.92E-03	0.92
GRM4	2914	-1.09	-1.32	-0.86	6.40E-05	3.92E-03	0.88
KRT19	3880	-1.07	-1.25	-0.90	1.91E-05	3.92E-03	0.83
MEIS1	4211	-1.23	-1.43	-1.02	2.22E-05	3.92E-03	0.89
STON1	11037	-1.23	-1.47	-0.98	5.37E-05	3.92E-03	0.84
RCAN3	11123	-1.32	-1.54	-1.09	2.19E-05	3.92E-03	0.91
RHBG	57127	-1.37	-1.65	-1.08	6.42E-05	3.92E-03	0.91
PCP2	126006	-1.56	-1.79	-1.34	1.01E-05	3.92E-03	0.96
C5orf38	153571	-1.24	-1.48	-0.99	4.72E-05	3.92E-03	0.9
IRX2	153572	-1.70	-2.02	-1.39	3.50E-05	3.92E-03	0.93
C19orf46	163183	-1.58	-1.91	-1.24	7.13E-05	3.92E-03	0.93
ADAMTS18	170692	-1.62	-1.85	-1.38	1.03E-05	3.92E-03	0.78
CRNDE	643911	-2.05	-2.47	-1.62	6.07E-05	3.92E-03	0.93
PGAM2	5224	-1.11	-1.35	-0.87	7.64E-05	3.98E-03	0.79
C7orf16	10842	-1.06	-1.29	-0.82	8.05E-05	3.99E-03	0.84
IRX5	10265	-1.66	-2.03	-1.29	8.44E-05	4.05E-03	0.94
LDLRAP1	26119	-1.06	-1.30	-0.82	8.63E-05	4.08E-03	0.79
IL28RA	163702	-1.18	-1.44	-0.91	9.28E-05	4.14E-03	0.78
CBLN1	869	-2.02	-2.48	-1.55	1.01E-04	4.19E-03	0.92
IL16	3603	-1.09	-1.34	-0.84	9.66E-05	4.19E-03	0.87
GPRIN2	9721	-1.40	-1.73	-1.07	1.09E-04	4.24E-03	0.92
CALB2	794	-1.06	-1.31	-0.81	1.11E-04	4.25E-03	0.86
EPB41	2035	-1.20	-1.48	-0.91	1.21E-04	4.25E-03	0.87
IRX3	79191	-1.55	-1.91	-1.18	1.15E-04	4.25E-03	0.93
SPINK6	404203	-1.20	-1.49	-0.91	1.26E-04	4.34E-03	0.92
ZNF521	25925	-1.05	-1.31	-0.79	1.43E-04	4.50E-03	0.9
KLHL1	57626	-1.04	-1.30	-0.79	1.44E-04	4.50E-03	0.84
DOK7	285489	-1.48	-1.85	-1.11	1.46E-04	4.50E-03	0.87
CNPY1	285888	-1.16	-1.45	-0.87	1.45E-04	4.50E-03	0.87
THRSP	7069	-1.20	-1.50	-0.89	1.63E-04	4.62E-03	0.83
ZIC3	7547	-1.41	-1.78	-1.05	1.68E-04	4.63E-03	0.88
STAC	6769	-1.20	-1.52	-0.88	2.02E-04	4.82E-03	0.86
TIMP4	7079	-1.13	-1.43	-0.83	2.02E-04	4.82E-03	0.81
PDZK1	5174	-1.18	-1.50	-0.86	2.18E-04	4.95E-03	0.88
LGR6	59352	-1.29	-1.66	-0.92	2.94E-04	5.43E-03	0.69
TFAP2B	7021	-1.66	-2.14	-1.18	2.94E-04	5.43E-03	0.95
CBLN3	643866	-1.24	-1.60	-0.88	3.15E-04	5.55E-03	0.9
LEMD1	93273	-1.14	-1.47	-0.80	3.19E-04	5.58E-03	0.79
EOMES	8320	-1.27	-1.65	-0.90	3.28E-04	5.59E-03	0.97
PIRT	644139	-1.14	-1.48	-0.80	3.48E-04	5.75E-03	0.88
MYT1	4661	-1.02	-1.33	-0.72	3.59E-04	5.80E-03	0.86
SLC35F4	341880	-1.27	-1.65	-0.89	3.60E-04	5.80E-03	0.85
CCDC155	147872	-1.29	-1.68	-0.90	3.64E-04	5.83E-03	0.97
C9orf171	389799	-1.29	-1.68	-0.90	3.67E-04	5.83E-03	0.79
BARHL1	56751	-1.47	-1.91	-1.02	3.70E-04	5.86E-03	0.87
UNCX	340260	-1.35	-1.76	-0.94	3.80E-04	5.92E-03	0.88
SLITRK6	84189	-1.54	-2.01	-1.07	3.84E-04	5.92E-03	0.73
GABRA6	2559	-1.53	-1.99	-1.06	3.94E-04	5.99E-03	0.96
ZIC4	84107	-1.66	-2.17	-1.15	3.97E-04	6.00E-03	0.91
EBF1	1879	-1.22	-1.60	-0.85	4.02E-04	6.03E-03	0.83
MAB21L1	4081	-1.30	-1.70	-0.90	4.07E-04	6.03E-03	0.9
CDH15	1013	-1.82	-2.39	-1.25	4.36E-04	6.20E-03	0.95
NUAK2	81788	-1.00	-1.32	-0.68	4.64E-04	6.33E-03	0.73
IRF6	3664	-1.03	-1.37	-0.70	5.15E-04	6.62E-03	0.91
ANGPTL7	10218	-1.03	-1.37	-0.70	5.20E-04	6.66E-03	0.87
BARHL2	343472	-1.24	-1.64	-0.83	5.22E-04	6.67E-03	0.89
CRTAM	56253	-1.66	-2.20	-1.12	5.39E-04	6.72E-03	0.95
SYT2	127833	-1.04	-1.39	-0.70	5.48E-04	6.77E-03	0.7
ZIC1	7545	-1.75	-2.33	-1.17	5.61E-04	6.82E-03	0.91
STK32A	202374	-1.02	-1.36	-0.67	6.81E-04	7.48E-03	0.74
PCSK9	255738	-1.29	-1.74	-0.84	7.12E-04	7.64E-03	0.86
ZP2	7783	-1.14	-1.53	-0.74	7.29E-04	7.70E-03	0.95
SLC22A31	146429	-1.09	-1.47	-0.71	7.57E-04	7.82E-03	0.88
CLCNKB	1188	-1.23	-1.68	-0.78	8.91E-04	8.40E-03	0.88
FLT3	2322	-1.10	-1.52	-0.67	1.17E-03	9.56E-03	0.82
DLK1	8788	-1.03	-1.46	-0.59	1.73E-03	1.18E-02	0.47
EBF3	253738	-1.12	-1.62	-0.63	2.10E-03	1.31E-02	0.86
FAT2	2196	-1.23	-1.79	-0.66	2.52E-03	1.45E-02	0.79
PVALB	5816	-1.26	-1.86	-0.65	3.01E-03	1.62E-02	0.89

Supplementary Table 3 Differentially downregulated genes in Network D (anterior cingulate network).

Gene name	Entrez ID	Fold-change	Lower95	Upper95	P-value	BH	Differential stability
CA12	771	-1.40	-1.69	-1.11	6.02E-05	6.39E-03	0.84
CHRNA3	1136	-1.33	-1.60	-1.05	6.41E-05	6.39E-03	0.84
DRD2	1813	-1.05	-1.19	-0.90	7.31E-06	6.39E-03	0.88
MAB21L1	4081	-1.06	-1.31	-0.82	9.63E-05	6.39E-03	0.9
PENK	5179	-1.11	-1.35	-0.86	7.67E-05	6.39E-03	0.81
STAC	6769	-1.32	-1.61	-1.03	8.59E-05	6.39E-03	0.86
LIPG	9388	-1.15	-1.42	-0.88	1.11E-04	6.39E-03	0.79
GPRIN2	9721	-1.34	-1.66	-1.03	1.15E-04	6.39E-03	0.92
RGS8	85397	-1.28	-1.55	-1.00	7.00E-05	6.39E-03	0.82
IRX2	153572	-1.96	-2.40	-1.51	9.72E-05	6.39E-03	0.93
C19orf46	163183	-1.40	-1.72	-1.09	9.22E-05	6.39E-03	0.93
DACT2	168002	-1.06	-1.26	-0.86	3.93E-05	6.39E-03	0.79
ANKRD34B	340120	-1.10	-1.34	-0.85	8.65E-05	6.39E-03	0.82
RSPO4	343637	-1.04	-1.25	-0.84	4.59E-05	6.39E-03	0.9
CRNDE	643911	-2.12	-2.52	-1.73	3.60E-05	6.39E-03	0.93
TMEM90A	646658	-1.13	-1.33	-0.93	3.08E-05	6.39E-03	0.85
IRX3	79191	-1.48	-1.84	-1.12	1.35E-04	6.54E-03	0.93
ECEL1	9427	-1.25	-1.57	-0.94	1.57E-04	6.76E-03	0.83
IRX5	10265	-1.72	-2.18	-1.27	1.87E-04	6.90E-03	0.94
ZIC4	84107	-1.20	-1.52	-0.88	2.09E-04	6.93E-03	0.91
SLC35F4	341880	-1.05	-1.34	-0.76	2.28E-04	7.13E-03	0.85
SLITRK6	84189	-1.34	-1.71	-0.97	2.44E-04	7.22E-03	0.73
C5orf38	153571	-1.49	-1.91	-1.07	2.62E-04	7.35E-03	0.9
CDH15	1013	-1.47	-1.91	-1.03	3.54E-04	7.95E-03	0.95
COL8A2	1296	-1.03	-1.34	-0.71	3.95E-04	8.20E-03	0.77
NTS	4922	-1.47	-1.93	-1.02	4.03E-04	8.23E-03	0.79
DLK1	8788	-1.13	-1.48	-0.77	4.77E-04	8.75E-03	0.47
DAO	1610	-2.06	-2.72	-1.40	4.94E-04	8.87E-03	0.92
WNT11	7481	-1.13	-1.49	-0.76	5.39E-04	9.08E-03	0.8
PIRT	644139	-1.01	-1.36	-0.67	6.54E-04	9.91E-03	0.88
C9orf171	389799	-1.32	-1.79	-0.84	8.43E-04	1.06E-02	0.79
TIMP4	7079	-1.02	-1.40	-0.65	8.71E-04	1.07E-02	0.81
STON1	11037	-1.13	-1.56	-0.70	1.11E-03	1.20E-02	0.84
TFAP2B	7021	-1.20	-1.68	-0.73	1.32E-03	1.30E-02	0.95
GBX2	2637	-1.12	-1.57	-0.66	1.45E-03	1.36E-02	0.67
PCP2	126006	-1.29	-1.83	-0.76	1.61E-03	1.42E-02	0.96
EBF3	253738	-1.06	-1.51	-0.60	1.86E-03	1.53E-02	0.86
ZIC1	7545	-1.04	-1.50	-0.58	2.12E-03	1.63E-02	0.91
BARHL1	56751	-1.04	-1.52	-0.56	2.65E-03	1.83E-02	0.87

Supplementary Table 4 Differentially upregulated genes in Network C (posterior cingulate network).

Gene name	Entrez ID	Fold-change	Lower95	Upper95	P-value	BH	Differential stability
ADRA1D	146	1.33	1.07	1.58	4.27E-05	3.92E-03	0.9
CAMK2A	815	1.95	1.62	2.27	2.08E-05	3.92E-03	0.93
CDH9	1007	1.68	1.42	1.94	1.48E-05	3.92E-03	0.92
CHN1	1123	1.13	0.90	1.36	5.88E-05	3.92E-03	0.93
EGR3	1960	2.03	1.66	2.39	3.14E-05	3.92E-03	0.93
EXTL1	2134	1.16	0.94	1.38	3.90E-05	3.92E-03	0.93
F12	2161	1.05	0.87	1.22	2.27E-05	3.92E-03	0.91
FHL2	2274	1.50	1.24	1.77	2.75E-05	3.92E-03	0.93
GABRA5	2558	1.98	1.62	2.34	3.17E-05	3.92E-03	0.94
GRIN2B	2904	1.21	0.98	1.44	4.07E-05	3.92E-03	0.81
HTR1A	3350	1.33	1.10	1.55	2.31E-05	3.92E-03	0.82
ITPKA	3706	1.34	1.09	1.59	3.78E-05	3.92E-03	0.95
LMO7	4008	1.00	0.80	1.20	4.84E-05	3.92E-03	0.89
NELL2	4753	1.01	0.83	1.18	2.48E-05	3.92E-03	0.87
NNMT	4837	1.39	1.14	1.64	3.00E-05	3.92E-03	0.84
NPTX2	4885	1.50	1.23	1.78	3.41E-05	3.92E-03	0.9

NRGN	4900	2.73	2.22	3.23	3.61E-05	3.92E-03	0.97
PCDH8	5100	1.70	1.39	2.01	3.30E-05	3.92E-03	0.92
PDE2A	5138	1.59	1.40	1.79	4.78E-06	3.92E-03	0.95
PSD	5662	1.02	0.88	1.17	1.07E-05	3.92E-03	0.86
RAB27B	5874	1.15	0.92	1.38	4.97E-05	3.92E-03	0.89
RASGRF2	5924	1.04	0.88	1.20	1.49E-05	3.92E-03	0.85
SLC30A3	7781	2.37	2.02	2.72	1.12E-05	3.92E-03	0.94
ENC1	8507	1.96	1.62	2.30	2.46E-05	3.92E-03	0.95
LMO4	8543	1.02	0.80	1.24	6.85E-05	3.92E-03	0.91
HRK	8739	1.27	1.09	1.44	8.33E-06	3.92E-03	0.91
KALRN	8997	1.13	0.98	1.27	5.77E-06	3.92E-03	0.88
LDB2	9079	1.73	1.45	2.01	1.71E-05	3.92E-03	0.94
LHX2	9355	2.21	1.82	2.60	2.86E-05	3.92E-03	0.94
CARTPT	9607	1.71	1.38	2.04	4.08E-05	3.92E-03	0.86
CACNG3	10368	1.52	1.22	1.83	5.19E-05	3.92E-03	0.89
PLK2	10769	1.48	1.24	1.72	1.81E-05	3.92E-03	0.95
LZTS1	11178	1.48	1.26	1.71	1.33E-05	3.92E-03	0.94
MAST3	23031	1.20	1.04	1.37	7.91E-06	3.92E-03	0.93
KCNH3	23416	1.11	0.93	1.30	1.93E-05	3.92E-03	0.93
RIMBP2	23504	1.03	0.89	1.17	7.36E-06	3.92E-03	0.91
MMD	23531	1.13	0.94	1.32	2.34E-05	3.92E-03	0.91
AK5	26289	1.61	1.28	1.95	5.90E-05	3.92E-03	0.95
SYT17	51760	1.08	0.86	1.30	5.34E-05	3.92E-03	0.86
SEMA5B	54437	1.04	0.88	1.19	1.24E-05	3.92E-03	0.83
FEZF2	55079	2.43	1.99	2.86	2.94E-05	3.92E-03	0.94
KCNQ5	56479	1.14	0.94	1.34	2.72E-05	3.92E-03	0.88
SLC17A7	57030	1.90	1.58	2.21	1.93E-05	3.92E-03	0.93
CAMK1G	57172	1.03	0.83	1.23	4.50E-05	3.92E-03	0.91
KIAA1324	57535	1.07	0.91	1.23	1.19E-05	3.92E-03	0.91
LRRC7	57554	1.66	1.37	1.95	2.62E-05	3.92E-03	0.95
CLSTN2	64084	1.02	0.83	1.21	3.43E-05	3.92E-03	0.86
PCDH20	64881	1.57	1.24	1.89	6.48E-05	3.92E-03	0.92
AC006273.1	79948	1.23	1.05	1.41	1.19E-05	3.92E-03	0.87
NETO1	81832	1.59	1.30	1.88	3.36E-05	3.92E-03	0.94
ST6GALNAC5	81849	1.58	1.35	1.80	9.67E-06	3.92E-03	0.84
SYT16	83851	1.08	0.88	1.27	3.05E-05	3.92E-03	0.85
NCALD	83988	1.10	0.91	1.29	2.40E-05	3.92E-03	0.91
SYDE2	84144	1.04	0.82	1.26	6.81E-05	3.92E-03	0.87
LRRC62	114794	1.05	0.90	1.21	1.03E-05	3.92E-03	0.9
CPNE4	131034	1.56	1.30	1.82	2.18E-05	3.92E-03	0.89
LOC158696	158696	1.13	0.96	1.31	1.51E-05	3.92E-03	0.87
KCNG3	170850	1.34	1.11	1.58	2.75E-05	3.92E-03	0.85
CREG2	200407	2.18	1.79	2.58	3.18E-05	3.92E-03	0.96
C8orf46	254778	1.09	0.95	1.24	7.27E-06	3.92E-03	0.9
CHSY3	337876	1.18	1.03	1.34	6.67E-06	3.92E-03	0.85
FAM19A2	338811	1.20	0.97	1.42	3.69E-05	3.92E-03	0.92
MYBPHL	343263	1.20	0.98	1.42	3.54E-05	3.92E-03	0.87
KCNT2	343450	1.01	0.85	1.17	1.50E-05	3.92E-03	0.87
C2orf55	343990	1.42	1.17	1.67	2.65E-05	3.92E-03	0.94
C1orf95	375057	1.08	0.88	1.28	3.67E-05	3.92E-03	0.72
KCTD4	386618	1.24	0.98	1.50	6.31E-05	3.92E-03	0.9
FAM19A1	407738	1.86	1.63	2.08	4.29E-06	3.92E-03	0.91
LOC440084	440084	1.06	0.85	1.26	4.47E-05	3.92E-03	0.85
CHRM3	1131	1.32	1.03	1.60	7.38E-05	3.94E-03	0.9
KIRREL2	84063	1.16	0.91	1.41	7.42E-05	3.94E-03	0.82
STX1A	6804	1.11	0.87	1.35	7.69E-05	3.98E-03	0.93
DLGAP2	9228	1.32	1.03	1.61	8.04E-05	3.99E-03	0.91
AKAP5	9495	1.34	1.05	1.64	7.93E-05	3.99E-03	0.9
TNFAIP8L3	388121	1.35	1.05	1.65	8.21E-05	4.01E-03	0.81
NEURL1B	54492	1.23	0.95	1.51	9.11E-05	4.14E-03	0.87
RP4-788L13.1	9890	1.04	0.80	1.28	9.80E-05	4.19E-03	0.81
KCNV1	27012	2.28	1.75	2.80	1.02E-04	4.19E-03	0.94
MKL2	57496	1.07	0.82	1.32	1.01E-04	4.19E-03	0.94
MUM1L1	139221	1.30	1.00	1.61	1.02E-04	4.19E-03	0.86
VIP	7432	1.67	1.28	2.06	1.06E-04	4.23E-03	0.83
LY6H	4062	1.61	1.23	1.98	1.08E-04	4.24E-03	0.93
CAMKV	79012	1.52	1.16	1.88	1.15E-04	4.25E-03	0.93
FOXG1B	2290	3.08	2.34	3.82	1.23E-04	4.28E-03	0.98
MPPED1	758	1.29	0.98	1.60	1.29E-04	4.37E-03	0.94
TBR1	10716	1.49	1.12	1.85	1.32E-04	4.38E-03	0.93
DACH2	117154	1.37	1.03	1.70	1.33E-04	4.41E-03	0.89
CCK	885	3.01	2.26	3.75	1.42E-04	4.50E-03	0.94
SCN3B	55800	1.19	0.89	1.48	1.43E-04	4.50E-03	0.85
KCTD16	57528	1.39	1.04	1.74	1.48E-04	4.50E-03	0.71
BAIAP3	8938	1.20	0.90	1.50	1.52E-04	4.52E-03	0.88
ANO3	63982	1.63	1.21	2.04	1.64E-04	4.63E-03	0.93
PPAPR5	163404	1.07	0.80	1.35	1.71E-04	4.65E-03	0.87
TMEM155	132332	2.80	2.07	3.52	1.79E-04	4.74E-03	0.95
RASAL1	8437	1.15	0.85	1.45	1.84E-04	4.77E-03	0.83
GABRA4	2557	1.13	0.83	1.43	1.94E-04	4.78E-03	0.9
ARC	23237	1.32	0.97	1.67	1.94E-04	4.78E-03	0.85
MOXD1	26002	2.02	1.49	2.56	1.89E-04	4.78E-03	0.9

NEUROD6	63974	1.90	1.40	2.40	1.95E-04	4.78E-03	0.88
KHDRBS2	202559	1.22	0.89	1.54	1.98E-04	4.81E-03	0.93
GPR26	2849	1.49	1.09	1.89	2.05E-04	4.87E-03	0.89
C2orf80	389073	1.23	0.90	1.56	2.06E-04	4.87E-03	0.93
HGF	3082	1.08	0.79	1.38	2.26E-04	4.97E-03	0.77
KIAA1239	57495	1.02	0.74	1.29	2.25E-04	4.97E-03	0.86
C14orf23	387978	1.61	1.18	2.05	2.22E-04	4.97E-03	0.94
ICAM5	7087	1.82	1.32	2.32	2.31E-04	4.98E-03	0.93
AP003108.2	390205	1.14	0.82	1.46	2.52E-04	5.16E-03	0.82
C1orf115	79762	1.21	0.87	1.55	2.61E-04	5.23E-03	0.93
EFNB3	1949	1.01	0.73	1.30	2.65E-04	5.25E-03	0.87
LINGO1	84894	1.06	0.76	1.36	2.85E-04	5.39E-03	0.85
MEF2C	4208	1.16	0.82	1.50	3.09E-04	5.50E-03	0.94
TMEM132B	114795	1.06	0.75	1.36	3.10E-04	5.51E-03	0.84
GDA	9615	2.68	1.89	3.47	3.26E-04	5.59E-03	0.95
ATRNL1	26033	1.18	0.84	1.53	3.20E-04	5.59E-03	0.91
OTX1	5013	1.14	0.80	1.48	3.37E-04	5.63E-03	0.83
THRB	7068	1.16	0.81	1.50	3.38E-04	5.63E-03	0.86
C6orf126	389383	1.10	0.78	1.43	3.38E-04	5.63E-03	0.8
FILIP1	27145	1.30	0.91	1.69	3.56E-04	5.79E-03	0.88
NR2E1	7101	1.38	0.96	1.79	3.73E-04	5.88E-03	0.89
NPY	4852	1.77	1.23	2.31	3.79E-04	5.92E-03	0.88
KLK7	5650	1.13	0.79	1.47	3.81E-04	5.92E-03	0.81
RGS14	10636	1.30	0.90	1.70	3.89E-04	5.95E-03	0.89
SSTR1	6751	1.02	0.71	1.33	3.96E-04	6.00E-03	0.88
FAM81A	145773	1.51	1.05	1.97	3.99E-04	6.01E-03	0.95
KCNS2	3788	1.11	0.77	1.45	4.05E-04	6.03E-03	0.86
NEK2	4751	1.08	0.75	1.41	4.10E-04	6.04E-03	0.77
RGS4	5999	1.44	1.00	1.89	4.15E-04	6.06E-03	0.95
NPTXR	23467	1.03	0.71	1.35	4.33E-04	6.18E-03	0.85
ZNF831	128611	1.60	1.10	2.11	4.41E-04	6.21E-03	0.88
OR14I1	401994	1.96	1.35	2.57	4.41E-04	6.21E-03	0.94
C1QL1	389941	1.44	0.99	1.89	4.48E-04	6.25E-03	0.84
EMX2OS	196047	1.37	0.94	1.80	4.62E-04	6.33E-03	0.87
HRH1	3269	1.27	0.87	1.68	4.65E-04	6.33E-03	0.91
WDR86	349136	1.08	0.74	1.43	4.74E-04	6.38E-03	0.8
EFCAB1	79645	1.09	0.74	1.44	4.79E-04	6.39E-03	0.78
SST	6750	1.76	1.19	2.34	5.25E-04	6.67E-03	0.92
KCNC2	3747	1.45	0.98	1.93	5.39E-04	6.72E-03	0.93
EGR2	1959	1.15	0.77	1.53	5.45E-04	6.76E-03	0.73
NPPA	4878	1.64	1.09	2.19	5.88E-04	6.98E-03	0.9
RBP4	5950	1.52	1.00	2.03	6.28E-04	7.17E-03	0.89
STYK1	55359	1.15	0.76	1.53	6.32E-04	7.17E-03	0.89
FAM148C	126567	1.01	0.67	1.35	6.37E-04	7.20E-03	0.91
KIF17	57576	1.05	0.69	1.41	6.53E-04	7.32E-03	0.86
HSPB3	8988	2.07	1.36	2.78	6.71E-04	7.42E-03	0.94
CDH8	1006	1.08	0.70	1.46	7.38E-04	7.74E-03	0.87
DLX6-AS1	285987	1.19	0.77	1.61	7.49E-04	7.79E-03	0.92
SERPINF1	5176	1.12	0.72	1.52	7.78E-04	7.88E-03	0.85
SLIT1	6585	1.59	1.02	2.15	7.97E-04	8.00E-03	0.86
CORT	1325	1.08	0.69	1.46	8.12E-04	8.05E-03	0.93
TNNT2	7139	1.33	0.84	1.81	8.95E-04	8.40E-03	0.9
DDN	23109	1.40	0.89	1.91	8.96E-04	8.40E-03	0.91
TAC3	6866	1.56	0.99	2.13	9.16E-04	8.48E-03	0.9
LMO3	55885	1.19	0.75	1.62	9.40E-04	8.58E-03	0.91
AC078937.4	286002	1.67	1.05	2.29	9.60E-04	8.67E-03	0.93
WIF1	11197	1.02	0.64	1.41	9.78E-04	8.74E-03	0.81
CCDC3	83643	1.04	0.65	1.43	1.02E-03	8.96E-03	0.91
FAM5B	57795	1.17	0.73	1.62	1.02E-03	8.96E-03	0.89
ADCY2	108	1.55	0.97	2.14	1.05E-03	9.04E-03	0.92
LYZL4	131375	1.04	0.65	1.43	1.06E-03	9.10E-03	0.87
LHX6	26468	1.79	1.11	2.48	1.07E-03	9.11E-03	0.93
ZNF727	442319	1.50	0.92	2.07	1.11E-03	9.25E-03	0.92
HTR2A	3356	1.38	0.84	1.91	1.18E-03	9.59E-03	0.92
PKD2L1	9033	1.49	0.91	2.07	1.19E-03	9.65E-03	0.92
DLX1	1745	1.57	0.96	2.19	1.25E-03	9.90E-03	0.94
C13orf36	400120	1.38	0.83	1.93	1.29E-03	1.01E-02	0.85
EMX2	2018	1.16	0.70	1.62	1.31E-03	1.01E-02	0.82
CXCL14	9547	1.15	0.69	1.61	1.35E-03	1.03E-02	0.9
KCNF1	3754	1.48	0.88	2.07	1.41E-03	1.05E-02	0.9
RASL10A	10633	1.12	0.67	1.57	1.43E-03	1.06E-02	0.83
LY86-AS1	285780	1.93	1.15	2.72	1.44E-03	1.06E-02	0.96
NGEF	25791	1.42	0.84	2.00	1.52E-03	1.10E-02	0.95
TM7SF4	81501	1.04	0.61	1.46	1.53E-03	1.10E-02	0.75
PPEF1	5475	1.13	0.67	1.60	1.55E-03	1.11E-02	0.85
TRIM54	57159	1.71	0.99	2.43	1.68E-03	1.16E-02	0.92
HS3ST2	9956	1.09	0.63	1.55	1.72E-03	1.18E-02	0.9
SATB2	23314	1.23	0.71	1.75	1.79E-03	1.21E-02	0.94
NECAB2	54550	1.18	0.68	1.68	1.80E-03	1.21E-02	0.89
SLC26A4	5172	1.34	0.76	1.92	1.91E-03	1.25E-02	0.92
PCSK1	5122	1.15	0.65	1.65	1.92E-03	1.25E-02	0.9
RPRML	388394	1.19	0.66	1.71	2.09E-03	1.31E-02	0.93

AC109486.1	25859	1.03	0.57	1.50	2.28E-03	1.37E-02	0.9
KCNS1	3787	1.54	0.84	2.24	2.35E-03	1.40E-02	0.94
KIAA0748	9840	1.17	0.63	1.70	2.47E-03	1.44E-02	0.93
AC002563.2	387890	1.28	0.69	1.87	2.55E-03	1.46E-02	0.89
MYB	4602	1.03	0.55	1.50	2.59E-03	1.47E-02	0.82
FREM3	166752	1.05	0.56	1.53	2.63E-03	1.49E-02	0.82
LOC646627	646627	1.73	0.93	2.53	2.64E-03	1.49E-02	0.92
AC079341.1	100192379	1.33	0.71	1.95	2.70E-03	1.51E-02	0.94
CRH	1392	1.19	0.63	1.75	2.78E-03	1.54E-02	0.87
THEMIS	387357	1.57	0.80	2.34	3.37E-03	1.73E-02	0.95
NEUROD2	4761	1.06	0.52	1.60	3.96E-03	1.91E-02	0.93
KCNJ4	3761	1.50	0.73	2.27	4.04E-03	1.93E-02	0.91
ZBBX	79740	1.06	0.52	1.60	4.07E-03	1.94E-02	0.88
GALNTL5	168391	1.40	0.68	2.13	4.23E-03	1.98E-02	0.94
ANXA8	653145	1.22	0.58	1.86	4.45E-03	2.04E-02	0.85
C6orf105	84830	1.08	0.51	1.65	4.65E-03	2.10E-02	0.91
RXFP1	59350	1.09	0.48	1.70	6.04E-03	2.48E-02	0.92
GAST	2520	1.25	0.53	1.97	6.46E-03	2.59E-02	0.77
GLP2R	9340	1.04	0.43	1.66	7.26E-03	2.79E-02	0.82
FAP	2191	1.02	0.38	1.67	9.73E-03	3.37E-02	0.86

Supplementary Table 5 Differentially upregulated genes in Network D (anterior cingulate network).

Gene name	Entrez ID	Fold-change	Lower95	Upper95	P-value	BH	Differential stability
ADRA1B	147	1.24	1.04	1.43	1.69E-05	6.39E-03	0.83
MPPED1	758	1.81	1.40	2.22	9.61E-05	6.39E-03	0.94
CCKBR	887	1.37	1.09	1.66	6.08E-05	6.39E-03	0.82
CDH13	1012	1.42	1.15	1.70	4.54E-05	6.39E-03	0.91
CENPF	1063	1.01	0.82	1.20	3.89E-05	6.39E-03	0.67
COL5A2	1290	1.54	1.22	1.86	6.45E-05	6.39E-03	0.85
CRYM	1428	1.66	1.39	1.92	1.72E-05	6.39E-03	0.93
FHL2	2274	1.56	1.23	1.90	7.07E-05	6.39E-03	0.93
HTR1A	3350	1.06	0.82	1.31	9.69E-05	6.39E-03	0.82
KCNC2	3747	1.64	1.29	1.99	7.42E-05	6.39E-03	0.93
MATK	4145	1.07	0.83	1.31	8.78E-05	6.39E-03	0.81
MEF2C	4208	1.75	1.33	2.16	1.16E-04	6.39E-03	0.94
NEUROD2	4761	1.63	1.37	1.88	1.43E-05	6.39E-03	0.93
NPPA	4878	2.09	1.68	2.50	4.84E-05	6.39E-03	0.9
NPTX1	4884	1.17	0.98	1.36	1.89E-05	6.39E-03	0.94
NPTX2	4885	1.28	1.02	1.54	5.42E-05	6.39E-03	0.9
SERPINF1	5176	1.50	1.23	1.77	3.04E-05	6.39E-03	0.85
VIT	5212	1.03	0.83	1.23	4.45E-05	6.39E-03	0.86
PPEF1	5475	1.79	1.40	2.18	7.95E-05	6.39E-03	0.85
KLK7	5650	1.39	1.16	1.62	1.99E-05	6.39E-03	0.81
RASGRF2	5924	1.00	0.77	1.23	9.96E-05	6.39E-03	0.85
RS1	6247	1.44	1.16	1.72	4.17E-05	6.39E-03	0.82
SLN	6588	2.32	1.82	2.82	7.47E-05	6.39E-03	0.88
NR2E1	7101	1.18	0.90	1.46	1.14E-04	6.39E-03	0.89
SLC30A3	7781	2.56	2.10	3.02	3.03E-05	6.39E-03	0.94
RASAL1	8437	1.11	0.87	1.35	7.39E-05	6.39E-03	0.83
DOC2A	8448	1.16	0.97	1.36	1.95E-05	6.39E-03	0.88
HSPB3	8988	2.91	2.32	3.51	5.64E-05	6.39E-03	0.94
DLGAP2	9228	1.35	1.05	1.65	8.55E-05	6.39E-03	0.91
GLP2R	9340	1.44	1.11	1.77	9.47E-05	6.39E-03	0.82
CABP1	9478	1.69	1.42	1.97	1.86E-05	6.39E-03	0.9
MAFB	9935	1.14	0.97	1.30	1.13E-05	6.39E-03	0.81
HS3ST2	9956	2.13	1.68	2.58	6.32E-05	6.39E-03	0.9
SATB2	23314	2.09	1.60	2.58	1.14E-04	6.39E-03	0.94
LRRC8B	23507	1.03	0.83	1.24	5.30E-05	6.39E-03	0.9
SOSTDC1	25928	1.14	0.90	1.38	6.81E-05	6.39E-03	0.73
ATRNL1	26033	1.12	0.91	1.33	3.58E-05	6.39E-03	0.91
TIAM2	26230	1.17	0.95	1.39	3.49E-05	6.39E-03	0.9
AK5	26289	1.09	0.83	1.35	1.15E-04	6.39E-03	0.95
KCNV1	27012	2.25	1.78	2.73	6.64E-05	6.39E-03	0.94
RND1	27289	1.06	0.83	1.28	6.50E-05	6.39E-03	0.79
ASB2	51676	1.98	1.55	2.41	7.66E-05	6.39E-03	0.88
BCL11A	53335	1.03	0.79	1.26	9.34E-05	6.39E-03	0.92
SHC3	53358	1.13	0.88	1.38	8.10E-05	6.39E-03	0.88
FEZF2	55079	2.10	1.61	2.59	1.06E-04	6.39E-03	0.94
SLC17A7	57030	1.88	1.58	2.17	1.53E-05	6.39E-03	0.93
CAMK1G	57172	1.03	0.80	1.27	1.00E-04	6.39E-03	0.91
DPP10	57628	1.08	0.88	1.28	3.36E-05	6.39E-03	0.78
FAM5B	57795	1.12	0.86	1.38	1.04E-04	6.39E-03	0.89
RXFP1	59350	2.09	1.60	2.57	1.08E-04	6.39E-03	0.92
C6orf105	84830	2.04	1.59	2.50	8.43E-05	6.39E-03	0.91
DUSP27	92235	1.53	1.19	1.87	8.06E-05	6.39E-03	0.84
SH2D1B	117157	1.40	1.10	1.70	7.03E-05	6.39E-03	0.77
MUCL1	118430	1.24	1.00	1.49	4.81E-05	6.39E-03	0.81

RHEBL1	121268	1.29	1.02	1.57	6.58E-05	6.39E-03	0.83
C13orf16	121793	1.55	1.25	1.85	4.51E-05	6.39E-03	0.9
LYZL4	131375	1.29	0.99	1.60	1.08E-04	6.39E-03	0.87
TMEM155	132332	3.53	2.85	4.21	4.14E-05	6.39E-03	0.95
RTN4RL1	146760	1.26	1.02	1.49	3.69E-05	6.39E-03	0.86
WDR16	146845	1.12	0.86	1.38	9.87E-05	6.39E-03	0.81
ARX	170302	1.09	0.91	1.27	2.21E-05	6.39E-03	0.9
ANKRD24	170961	1.01	0.79	1.23	8.32E-05	6.39E-03	0.84
DHRS7C	201140	1.27	1.02	1.52	4.53E-05	6.39E-03	0.73
LY86-AS1	285780	2.80	2.30	3.30	2.98E-05	6.39E-03	0.96
FAM19A2	338811	1.35	1.05	1.65	8.78E-05	6.39E-03	0.92
LOC339524	339524	1.40	1.15	1.64	2.69E-05	6.39E-03	0.8
C2orf55	343990	1.52	1.16	1.87	1.09E-04	6.39E-03	0.94
LCE3C	353144	1.01	0.80	1.22	6.51E-05	6.39E-03	0.83
THEMIS	387357	2.83	2.17	3.50	1.10E-04	6.39E-03	0.95
AC002563.2	387890	2.28	1.95	2.62	1.17E-05	6.39E-03	0.89
C6orf126	389383	1.37	1.09	1.66	6.05E-05	6.39E-03	0.8
C1QL1	389941	1.83	1.44	2.23	7.28E-05	6.39E-03	0.84
KRT16P2	400578	1.48	1.21	1.74	2.93E-05	6.39E-03	0.88
AC112641.2	401097	1.59	1.27	1.91	5.43E-05	6.39E-03	0.9
ZNF727	442319	1.80	1.43	2.18	6.06E-05	6.39E-03	0.92
LOC643750	643750	1.01	0.85	1.18	1.91E-05	6.39E-03	0.74
KCNS1	3787	2.59	1.97	3.22	1.22E-04	6.44E-03	0.94
EPHX4	253152	1.02	0.78	1.27	1.22E-04	6.44E-03	0.87
LOC100287347	100287347	1.15	0.88	1.43	1.21E-04	6.44E-03	0.92
CCK	885	3.00	2.27	3.73	1.29E-04	6.46E-03	0.94
LHX2	9355	2.00	1.52	2.48	1.26E-04	6.46E-03	0.94
GDA	9615	2.83	2.15	3.52	1.28E-04	6.46E-03	0.95
AC078937.4	286002	2.28	1.73	2.83	1.26E-04	6.46E-03	0.93
CASQ1	844	1.53	1.16	1.90	1.30E-04	6.46E-03	0.83
KIAA0748	9840	2.44	1.85	3.03	1.30E-04	6.46E-03	0.93
TNNT2	7139	2.35	1.78	2.92	1.32E-04	6.47E-03	0.9
CREG2	200407	2.07	1.57	2.58	1.36E-04	6.54E-03	0.96
DACH2	117154	1.03	0.77	1.28	1.41E-04	6.55E-03	0.89
SLC26A4	5172	1.83	1.38	2.29	1.42E-04	6.56E-03	0.92
CDH9	1007	1.47	1.11	1.84	1.45E-04	6.64E-03	0.92
CYP26A1	1592	1.69	1.27	2.11	1.50E-04	6.64E-03	0.83
RASL11B	65997	1.20	0.90	1.50	1.50E-04	6.64E-03	0.91
SGK493	91461	1.31	0.98	1.63	1.52E-04	6.69E-03	0.82
NPFFR2	10886	1.24	0.93	1.55	1.53E-04	6.70E-03	0.76
ADCY2	108	1.91	1.42	2.39	1.61E-04	6.76E-03	0.92
EPHB6	2051	1.45	1.09	1.82	1.57E-04	6.76E-03	0.89
EXTL1	2134	1.15	0.86	1.44	1.64E-04	6.76E-03	0.93
LMO4	8543	1.37	1.02	1.72	1.62E-04	6.76E-03	0.91
KCNH3	23416	1.10	0.82	1.38	1.59E-04	6.76E-03	0.93
OVOL2	58495	1.70	1.27	2.13	1.58E-04	6.76E-03	0.89
NEUROD6	63974	1.92	1.43	2.40	1.58E-04	6.76E-03	0.88
OSBPL3	26031	1.22	0.91	1.54	1.66E-04	6.80E-03	0.84
NNMT	4837	1.23	0.91	1.54	1.67E-04	6.81E-03	0.84
RGS4	5999	1.92	1.43	2.42	1.71E-04	6.83E-03	0.95
AC010087.3	129293	1.40	1.04	1.76	1.76E-04	6.89E-03	0.83
RSPO2	340419	1.73	1.28	2.18	1.80E-04	6.89E-03	0.89
VIP	7432	1.85	1.36	2.33	1.83E-04	6.89E-03	0.83
VSNL1	7447	1.01	0.74	1.27	1.84E-04	6.89E-03	0.84
NMU	10874	1.47	1.09	1.85	1.85E-04	6.89E-03	0.82
AC079341.1	100192379	2.18	1.61	2.74	1.83E-04	6.89E-03	0.94
OLFM1	10439	1.04	0.76	1.31	1.89E-04	6.90E-03	0.93
ST6GALNAC5	81849	1.21	0.89	1.53	1.89E-04	6.90E-03	0.84
CRYBB1	1414	1.09	0.80	1.37	1.91E-04	6.90E-03	0.74
C6orf142	90523	1.83	1.34	2.31	1.94E-04	6.90E-03	0.88
KCNS2	3788	1.29	0.95	1.63	1.94E-04	6.91E-03	0.86
RBP4	5950	1.50	1.10	1.90	1.96E-04	6.91E-03	0.89
MCHR2	84539	1.71	1.26	2.16	1.95E-04	6.91E-03	0.93
MUM1L1	139221	1.53	1.12	1.94	2.00E-04	6.91E-03	0.86
GALNTL5	168391	2.08	1.53	2.64	2.00E-04	6.91E-03	0.94
DLX6-AS1	285987	1.32	0.97	1.67	1.98E-04	6.91E-03	0.92
PTGS2	5743	1.25	0.92	1.59	2.10E-04	6.93E-03	0.85
LMO3	55885	1.17	0.86	1.49	2.10E-04	6.93E-03	0.91
FAM162B	221303	1.10	0.80	1.39	2.07E-04	6.93E-03	0.86
C2orf80	389073	1.10	0.81	1.40	2.09E-04	6.93E-03	0.93
SOHLH1	402381	1.53	1.12	1.94	2.06E-04	6.93E-03	0.88
ENC1	8507	1.83	1.34	2.33	2.21E-04	7.11E-03	0.95
MAPK13	5603	1.00	0.73	1.28	2.22E-04	7.11E-03	0.78
C17orf96	100170841	1.55	1.13	1.97	2.22E-04	7.11E-03	0.93
AQP9	366	1.47	1.07	1.87	2.24E-04	7.13E-03	0.85
CHN1	1123	1.13	0.82	1.43	2.31E-04	7.13E-03	0.93
FOXG1B	2290	2.77	2.01	3.53	2.29E-04	7.13E-03	0.98
CACNG3	10368	1.55	1.13	1.97	2.25E-04	7.13E-03	0.89
POU6F2	11281	1.09	0.79	1.39	2.28E-04	7.13E-03	0.77
ABCC12	94160	1.77	1.28	2.25	2.30E-04	7.13E-03	0.9
PKD2L1	9033	2.11	1.53	2.69	2.35E-04	7.19E-03	0.92
GABRA5	2558	1.41	1.02	1.79	2.36E-04	7.19E-03	0.94

MYO5B	4645	1.10	0.79	1.40	2.40E-04	7.21E-03	0.76
MCHR1	2847	1.29	0.93	1.65	2.44E-04	7.22E-03	0.77
AC109486.1	25859	2.01	1.45	2.57	2.47E-04	7.26E-03	0.9
GPR26	2849	1.82	1.31	2.33	2.50E-04	7.27E-03	0.89
LDB2	9079	1.43	1.03	1.83	2.51E-04	7.27E-03	0.94
OR14I1	401994	2.08	1.50	2.66	2.57E-04	7.31E-03	0.94
HTR2A	3356	1.94	1.39	2.48	2.62E-04	7.35E-03	0.92
MOXD1	26002	1.57	1.12	2.01	2.68E-04	7.39E-03	0.9
BAIAP3	8938	1.20	0.86	1.54	2.72E-04	7.42E-03	0.88
NRGN	4900	2.61	1.87	3.36	2.76E-04	7.44E-03	0.97
RPRM	56475	1.29	0.92	1.66	2.77E-04	7.44E-03	0.84
STX1A	6804	1.42	1.01	1.82	2.82E-04	7.46E-03	0.93
EGR3	1960	1.69	1.20	2.17	2.88E-04	7.52E-03	0.93
DLX1	1745	1.91	1.36	2.46	2.91E-04	7.54E-03	0.94
TMEM132D	121256	1.26	0.90	1.62	2.94E-04	7.55E-03	0.87
PCDH8	5100	1.30	0.93	1.68	2.99E-04	7.58E-03	0.92
TBR1	10716	1.59	1.13	2.05	3.07E-04	7.62E-03	0.93
NUDT4P1	11163	1.19	0.85	1.54	3.09E-04	7.63E-03	0.79
KIAA1239	57495	1.43	1.02	1.85	3.14E-04	7.65E-03	0.86
NEK2	4751	1.23	0.87	1.59	3.29E-04	7.74E-03	0.77
KCNT2	343450	1.02	0.72	1.33	3.40E-04	7.85E-03	0.87
CRHBP	1393	1.26	0.88	1.63	3.43E-04	7.87E-03	0.86
ITPKA	3706	1.48	1.04	1.92	3.51E-04	7.93E-03	0.95
DNAJC5G	285126	1.02	0.72	1.33	3.53E-04	7.95E-03	0.82
WIF1	11197	1.03	0.72	1.34	3.55E-04	7.95E-03	0.81
KCNJ4	3761	1.73	1.21	2.25	3.61E-04	7.95E-03	0.91
AC005551.1	284422	1.10	0.77	1.43	3.66E-04	7.96E-03	0.7
HRH1	3269	1.56	1.09	2.04	3.69E-04	7.99E-03	0.91
PDE2A	5138	1.30	0.90	1.69	3.76E-04	8.08E-03	0.95
GUCA1B	2979	1.15	0.80	1.50	3.81E-04	8.15E-03	0.69
STYK1	55359	1.19	0.83	1.55	3.82E-04	8.15E-03	0.89
CDKL1	8814	1.00	0.69	1.31	4.15E-04	8.35E-03	0.87
KMO	8564	1.26	0.87	1.65	4.22E-04	8.41E-03	0.89
RASL10A	10633	1.30	0.90	1.71	4.31E-04	8.45E-03	0.83
CAMK2A	815	1.61	1.10	2.12	4.72E-04	8.72E-03	0.93
DDN	23109	1.21	0.82	1.60	4.84E-04	8.79E-03	0.91
ERICH1-AS1	619343	1.06	0.72	1.39	4.87E-04	8.83E-03	0.89
GYPE	2996	1.24	0.84	1.64	4.91E-04	8.87E-03	0.72
LOC100290023	100290023	1.45	0.98	1.91	4.92E-04	8.87E-03	0.84
MEPE	56955	1.07	0.73	1.42	5.04E-04	8.96E-03	0.74
SLCO2A1	6578	1.11	0.75	1.47	5.12E-04	8.99E-03	0.75
KCNQ5	56479	1.03	0.69	1.36	5.16E-04	8.99E-03	0.88
AC116165.2	283767	1.24	0.84	1.65	5.15E-04	8.99E-03	0.69
SCARA5	286133	1.34	0.91	1.78	5.16E-04	8.99E-03	0.73
FAM148C	126567	1.22	0.82	1.61	5.17E-04	8.99E-03	0.91
SLIT1	6585	1.28	0.86	1.70	5.33E-04	9.06E-03	0.86
MEIS3P2	56917	1.23	0.83	1.63	5.41E-04	9.08E-03	0.81
LRRTM4	80059	1.14	0.77	1.52	5.46E-04	9.12E-03	0.86
LY6H	4062	1.10	0.74	1.47	5.50E-04	9.14E-03	0.93
CHRM3	1131	1.57	1.05	2.09	5.54E-04	9.19E-03	0.9
TOX	9760	1.03	0.69	1.37	5.60E-04	9.25E-03	0.81
CRLF1	9244	1.16	0.77	1.54	5.65E-04	9.27E-03	0.83
FMN1	342184	1.15	0.77	1.53	5.76E-04	9.32E-03	0.86
NETO1	81832	1.33	0.89	1.78	5.82E-04	9.36E-03	0.94
ZBBX	79740	1.40	0.93	1.87	6.00E-04	9.52E-03	0.88
FREM3	166752	2.22	1.48	2.96	6.03E-04	9.53E-03	0.82
B3GALT2	8707	1.01	0.67	1.35	6.07E-04	9.54E-03	0.88
ANKRD56	345079	1.77	1.17	2.36	6.09E-04	9.57E-03	0.85
KHDRBS2	202559	1.37	0.91	1.83	6.14E-04	9.60E-03	0.93
C13orf39	196541	1.05	0.70	1.40	6.16E-04	9.61E-03	0.75
TYRP1	7306	1.43	0.94	1.92	6.39E-04	9.82E-03	0.85
TRIM54	57159	2.21	1.46	2.96	6.48E-04	9.89E-03	0.92
HGF	3082	1.29	0.85	1.73	6.77E-04	9.93E-03	0.77
NPY	4852	1.86	1.22	2.50	6.59E-04	9.93E-03	0.88
ICAM5	7087	1.52	0.99	2.04	6.76E-04	9.93E-03	0.93
NTN4	59277	1.05	0.69	1.42	6.64E-04	9.93E-03	0.87
CRH	1392	1.50	0.98	2.01	6.80E-04	9.94E-03	0.87
BAIAP2L2	80115	1.12	0.74	1.51	6.80E-04	9.94E-03	0.87
FAP	2191	1.16	0.76	1.57	6.93E-04	9.99E-03	0.86
C14orf23	387978	1.18	0.77	1.59	6.93E-04	9.99E-03	0.94
KCNF1	3754	1.54	1.00	2.07	7.06E-04	1.01E-02	0.9
COL24A1	255631	1.15	0.75	1.55	7.14E-04	1.01E-02	0.82
TAC3	6866	1.83	1.19	2.47	7.26E-04	1.02E-02	0.9
ANO3	63982	1.23	0.80	1.66	7.38E-04	1.02E-02	0.93
THEM5	284486	1.05	0.67	1.42	7.80E-04	1.04E-02	0.47
PDZRN3	23024	1.19	0.77	1.62	7.84E-04	1.05E-02	0.85
CXCL14	9547	1.08	0.70	1.47	7.95E-04	1.05E-02	0.9
NGEF	25791	1.66	1.06	2.25	8.13E-04	1.06E-02	0.95
DLX2	1746	1.21	0.78	1.64	8.16E-04	1.06E-02	0.89
SLC22A9	114571	1.27	0.81	1.73	8.30E-04	1.06E-02	0.85
TC2N	123036	1.11	0.71	1.51	8.22E-04	1.06E-02	0.78
PCSK1	5122	1.57	1.00	2.13	8.42E-04	1.06E-02	0.9

C1orf115	79762	1.27	0.81	1.73	8.39E-04	1.06E-02	0.93
ZBTB16	7704	1.01	0.65	1.38	8.58E-04	1.07E-02	0.74
OPN3	23596	1.25	0.80	1.71	8.61E-04	1.07E-02	0.86
FAM19A1	407738	1.25	0.80	1.71	8.65E-04	1.07E-02	0.91
KIF17	57576	1.16	0.74	1.58	8.87E-04	1.09E-02	0.86
LOC646627	646627	2.04	1.29	2.78	9.04E-04	1.10E-02	0.92
STEAP1	26872	1.11	0.71	1.52	9.08E-04	1.10E-02	0.76
ANXA8	653145	1.71	1.08	2.34	9.18E-04	1.10E-02	0.85
GAST	2520	1.22	0.77	1.67	9.42E-04	1.11E-02	0.77
SSTR1	6751	1.04	0.66	1.42	9.48E-04	1.12E-02	0.88
LHX6	26468	1.80	1.13	2.47	9.74E-04	1.13E-02	0.93
PRSS16	10279	1.13	0.71	1.55	9.89E-04	1.13E-02	0.78
RPRML	388394	1.52	0.95	2.09	9.87E-04	1.13E-02	0.93
PHLDA2	7262	1.02	0.64	1.41	1.02E-03	1.15E-02	0.85
C13orf36	400120	1.55	0.96	2.13	1.05E-03	1.17E-02	0.85
AC017096.1	150538	1.50	0.93	2.07	1.07E-03	1.18E-02	0.89
ANKRD62	342850	1.02	0.63	1.42	1.09E-03	1.20E-02	0.78
CORT	1325	1.10	0.68	1.51	1.10E-03	1.20E-02	0.93
CIDEA	1149	1.13	0.69	1.57	1.17E-03	1.23E-02	0.85
RTP1	132112	1.81	1.11	2.52	1.18E-03	1.23E-02	0.81
FAM81A	145773	1.34	0.82	1.86	1.20E-03	1.23E-02	0.95
NOS2	4843	1.01	0.62	1.41	1.22E-03	1.25E-02	0.83
EMX2	2018	1.07	0.65	1.49	1.25E-03	1.26E-02	0.82
ASGR2	433	1.12	0.68	1.57	1.31E-03	1.30E-02	0.76
IL12RB2	3595	1.04	0.62	1.45	1.33E-03	1.31E-02	0.61
SST	6750	1.60	0.97	2.24	1.33E-03	1.31E-02	0.92
FRMPD2	143162	1.60	0.96	2.24	1.34E-03	1.31E-02	0.82
PCDH20	64881	1.06	0.64	1.49	1.34E-03	1.31E-02	0.92
C8orf4	56892	1.01	0.60	1.41	1.42E-03	1.35E-02	0.69
C21orf128	150147	1.46	0.87	2.06	1.41E-03	1.35E-02	0.9
CAMKV	79012	1.18	0.70	1.65	1.44E-03	1.35E-02	0.93
GSG1	83445	1.38	0.81	1.94	1.51E-03	1.38E-02	0.6
GFRA2	2675	1.13	0.67	1.59	1.53E-03	1.39E-02	0.86
RORB	6096	1.19	0.70	1.67	1.53E-03	1.39E-02	0.88
EMX2OS	196047	1.19	0.70	1.69	1.53E-03	1.39E-02	0.87
HTR1F	3355	1.09	0.64	1.54	1.61E-03	1.42E-02	0.85
KCNH5	27133	1.34	0.79	1.90	1.61E-03	1.42E-02	0.88
CCDC3	83643	1.05	0.61	1.48	1.63E-03	1.43E-02	0.91
ZNF831	128611	1.08	0.62	1.53	1.76E-03	1.49E-02	0.88
GRASP	160622	1.09	0.63	1.56	1.77E-03	1.49E-02	0.89
ARC	23237	1.13	0.65	1.61	1.78E-03	1.50E-02	0.85
LZTS1	11178	1.08	0.62	1.54	1.85E-03	1.53E-02	0.94
AKAP5	9495	1.05	0.60	1.51	1.86E-03	1.53E-02	0.9
PAH	5053	1.19	0.67	1.71	1.97E-03	1.58E-02	0.72
GRHL2	79977	1.15	0.63	1.67	2.40E-03	1.75E-02	0.82
IGFBP2	3485	1.18	0.64	1.72	2.50E-03	1.78E-02	0.72
NCALD	83988	1.11	0.60	1.62	2.60E-03	1.81E-02	0.91
CBLN4	140689	1.03	0.55	1.51	2.63E-03	1.82E-02	0.77
FBXO40	51725	1.42	0.69	2.14	3.95E-03	2.28E-02	0.79
KCTD16	57528	1.16	0.56	1.75	4.10E-03	2.34E-02	0.71
IQGAP3	128239	1.35	0.64	2.07	4.51E-03	2.46E-02	0.74

Supplementary Table 6 Pathway analysis of differentially upregulated genes in Network C (posterior cingulate network) and Network D (anterior cingulate network). Many pathways are shared between both networks (red text).

Pathway	BH	Gene count
**Network C**		
Neuronal System	3.19E-09	23
Voltage gated Potassium channels	1.44E-06	8
Transmission across Chemical Synapses	6.95E-06	15
Neurotransmitter receptors and postsynaptic signal transmission	8.32E-06	13
GPCR ligand binding	2.16E-05	18
Class A/1 (Rhodopsin-like receptors)	2.42E-05	15
Potassium Channels	2.65E-05	9
Transcriptional Regulation by *MECP2*	1.45E-03	6
Amine ligand-binding receptors	1.93E-03	5
Ras activation upon Ca2+ influx through NMDA receptor	2.03E-03	4
Long-term potentiation	3.12E-03	4
CREB1 phosphorylation through NMDA receptor-mediated activation of RAS signaling	5.96E-03	4
Activation of NMDA receptors and postsynaptic events	7.18E-03	6
G alpha (q) signalling events	7.26E-03	9
Lysosphingolipid and LPA receptors	7.30E-03	3
Assembly and cell surface presentation of NMDA receptors	1.13E-02	4
Post NMDA receptor activation events	2.15E-02	5
Unblocking of NMDA receptors, glutamate binding and activation	2.62E-02	3
Negative regulation of NMDA receptor-mediated neuronal transmission	2.62E-02	3
G alpha (i) signalling events	2.86E-02	11
GABA receptor activation	3.67E-02	4
Peptide ligand-binding receptors	3.67E-02	7
Trafficking of AMPA receptors	4.97E-02	3
Glutamate binding, activation of AMPA receptors and synaptic plasticity	4.97E-02	3
**Network D**		
GPCR ligand binding	3.34E-06	23
Class A/1 (Rhodopsin-like receptors)	3.34E-06	19
Neuronal System	6.21E-06	21
Voltage gated Potassium channels	6.21E-06	8
G alpha (q) signalling events	2.61E-04	13
Peptide ligand-binding receptors	2.61E-04	12
Potassium Channels	2.61E-04	9
Amine ligand-binding receptors	6.30E-04	6
G alpha (i) signalling events	1.73E-03	16
Transmission across Chemical Synapses	6.93E-03	12
Neurotransmitter receptors and postsynaptic signal transmission	9.83E-03	10
Serotonin receptors	1.35E-02	3
Transcriptional Regulation by *MECP2*	3.56E-02	5

Supplementary Table 7 GO enrichment analysis of differentially upregulated genes in Network C (posterior cingulate network).

ID	Description	BH	Count
GO:0007611	learning or memory	8.20E-10	21
GO:0050804	modulation of chemical synaptic transmission	1.22E-09	26
GO:0099177	regulation of trans-synaptic signaling	1.22E-09	26
GO:0050890	cognition	3.36E-09	21
GO:0048167	regulation of synaptic plasticity	7.25E-08	16
GO:0008306	associative learning	1.60E-07	11
GO:0006813	potassium ion transport	8.74E-07	16
GO:0050806	positive regulation of synaptic transmission	1.03E-06	14
GO:0007612	learning	1.13E-06	13
GO:0099601	regulation of neurotransmitter receptor activity	1.40E-06	10

GO:0071805	potassium ion transmembrane transport	7.67E-06	14
GO:0007613	memory	1.09E-05	11
GO:0007187	G protein-coupled receptor signaling pathway, coupled to cyclic nucleotide second messenger	1.16E-05	15
GO:0010469	regulation of signaling receptor activity	1.51E-05	12
GO:0034765	regulation of ion transmembrane transport	1.74E-05	20
GO:0001662	behavioral fear response	2.19E-05	7
GO:0002209	behavioral defense response	2.47E-05	7
GO:0050803	regulation of synapse structure or activity	2.65E-05	14
GO:0042596	fear response	2.65E-05	7
GO:1900449	regulation of glutamate receptor signaling pathway	3.18E-05	8
GO:0031644	regulation of nervous system process	4.63E-05	11
GO:0050769	positive regulation of neurogenesis	5.01E-05	19
GO:0030900	forebrain development	7.56E-05	17
GO:0050807	regulation of synapse organization	7.56E-05	13
GO:0007215	glutamate receptor signaling pathway	9.42E-05	9
GO:0045666	positive regulation of neuron differentiation	1.46E-04	16
GO:2001257	regulation of cation channel activity	2.03E-04	11
GO:0021877	forebrain neuron fate commitment	2.03E-04	4
GO:0032412	regulation of ion transmembrane transporter activity	2.22E-04	13
GO:0021872	forebrain generation of neurons	2.43E-04	7
GO:0060291	long-term synaptic potentiation	2.60E-04	8
GO:0022898	regulation of transmembrane transporter activity	3.22E-04	13
GO:0051952	regulation of amine transport	3.70E-04	8
GO:0031646	positive regulation of nervous system process	3.70E-04	7
GO:0032409	regulation of transporter activity	4.77E-04	13
GO:0043266	regulation of potassium ion transport	5.53E-04	8
GO:0015837	amine transport	5.80E-04	8
GO:1903524	positive regulation of blood circulation	6.59E-04	7
GO:0021543	pallium development	8.06E-04	10
GO:0021879	forebrain neuron differentiation	8.06E-04	6
GO:0033555	multicellular organismal response to stress	8.75E-04	7
GO:0051260	protein homooligomerization	9.35E-04	10
GO:0021542	dentate gyrus development	1.09E-03	4
GO:0007188	adenylate cyclase-modulating G protein-coupled receptor signaling pathway	1.25E-03	11
GO:0050433	regulation of catecholamine secretion	1.25E-03	6
GO:0051961	negative regulation of nervous system development	1.34E-03	13
GO:0051259	protein complex oligomerization	1.41E-03	11
GO:0050432	catecholamine secretion	1.41E-03	6
GO:0015844	monoamine transport	1.41E-03	7
GO:0032094	response to food	2.21E-03	5
GO:1904062	regulation of cation transmembrane transport	2.30E-03	13
GO:0019932	second-messenger-mediated signaling	3.32E-03	15
GO:0060996	dendritic spine development	3.53E-03	7
GO:0021537	telencephalon development	3.93E-03	11
GO:0010769	regulation of cell morphogenesis involved in differentiation	4.38E-03	12
GO:1905809	negative regulation of synapse organization	4.57E-03	4
GO:0051937	catecholamine transport	4.59E-03	6
GO:0045665	negative regulation of neuron differentiation	5.06E-03	10
GO:0021895	cerebral cortex neuron differentiation	5.09E-03	4
GO:0060998	regulation of dendritic spine development	5.09E-03	6
GO:0008542	visual learning	5.13E-03	5
GO:0060078	regulation of postsynaptic membrane potential	5.24E-03	8
GO:0021761	limbic system development	5.48E-03	7
GO:0010460	positive regulation of heart rate	5.53E-03	4
GO:0021953	central nervous system neuron differentiation	6.28E-03	9
GO:0021892	cerebral cortex GABAergic interneuron differentiation	6.45E-03	3
GO:0050808	synapse organization	6.46E-03	14
GO:0050773	regulation of dendrite development	6.65E-03	8
GO:0010721	negative regulation of cell development	6.67E-03	12
GO:1901379	regulation of potassium ion transmembrane transport	7.19E-03	6
GO:0021987	cerebral cortex development	7.54E-03	7
GO:0007632	visual behavior	7.54E-03	5
GO:0050768	negative regulation of neurogenesis	7.54E-03	11
GO:1903522	regulation of blood circulation	7.54E-03	11

GO:0007193	adenylate cyclase-inhibiting G protein-coupled receptor signaling pathway	7.67E-03	6
GO:0016358	dendrite development	7.78E-03	10
GO:0033605	positive regulation of catecholamine secretion	9.44E-03	3
GO:1901016	regulation of potassium ion transmembrane transporter activity	1.01E-02	5
GO:0098664	G protein-coupled serotonin receptor signaling pathway	1.03E-02	4
GO:0021885	forebrain cell migration	1.06E-02	5
GO:0045907	positive regulation of vasoconstriction	1.12E-02	4
GO:0097154	GABAergic neuron differentiation	1.12E-02	3
GO:0046879	hormone secretion	1.19E-02	11
GO:0048814	regulation of dendrite morphogenesis	1.20E-02	6
GO:0046883	regulation of hormone secretion	1.23E-02	10
GO:0019935	cyclic-nucleotide-mediated signaling	1.31E-02	9
GO:1900451	positive regulation of glutamate receptor signaling pathway	1.31E-02	3
GO:2000310	regulation of NMDA receptor activity	1.31E-02	4
GO:0009914	hormone transport	1.42E-02	11
GO:0007210	serotonin receptor signaling pathway	1.43E-02	4
GO:0042391	regulation of membrane potential	1.60E-02	13
GO:0035296	regulation of tube diameter	1.65E-02	7
GO:0097746	regulation of blood vessel diameter	1.65E-02	7
GO:0048663	neuron fate commitment	1.65E-02	5
GO:0051954	positive regulation of amine transport	1.67E-02	4
GO:0035150	regulation of tube size	1.68E-02	7
GO:0022604	regulation of cell morphogenesis	1.70E-02	14
GO:0098962	regulation of postsynaptic neurotransmitter receptor activity	1.71E-02	3
GO:0003018	vascular process in circulatory system	1.73E-02	8
GO:0060079	excitatory postsynaptic potential	1.74E-02	6
GO:0045823	positive regulation of heart contraction	1.91E-02	4
GO:0060041	retina development in camera-type eye	2.20E-02	7
GO:0032095	regulation of response to food	2.26E-02	3
GO:1902259	regulation of delayed rectifier potassium channel activity	2.26E-02	3
GO:0003002	regionalization	2.47E-02	11
GO:0099565	chemical synaptic transmission, postsynaptic	2.56E-02	6
GO:0015850	organic hydroxy compound transport	2.83E-02	9
GO:0042310	vasoconstriction	2.90E-02	5
GO:1903532	positive regulation of secretion by cell	2.94E-02	10
GO:0061001	regulation of dendritic spine morphogenesis	2.98E-02	4
GO:0071277	cellular response to calcium ion	3.34E-02	5
GO:0021766	hippocampus development	3.42E-02	5
GO:0021954	central nervous system neuron development	3.42E-02	5
GO:0097061	dendritic spine organization	3.42E-02	5
GO:0001764	neuron migration	3.42E-02	7
GO:0007409	axonogenesis	3.53E-02	13
GO:0010447	response to acidic pH	3.53E-02	3
GO:0032098	regulation of appetite	3.53E-02	3
GO:0097756	negative regulation of blood vessel diameter	3.67E-02	5
GO:0048013	ephrin receptor signaling pathway	3.84E-02	5
GO:0021871	forebrain regionalization	3.87E-02	3
GO:2000311	regulation of AMPA receptor activity	3.87E-02	3
GO:0035418	protein localization to synapse	3.95E-02	5
GO:0010959	regulation of metal ion transport	3.96E-02	11
GO:0048168	regulation of neuronal synaptic plasticity	4.15E-02	4
GO:0032104	regulation of response to extracellular stimulus	4.20E-02	3
GO:0032107	regulation of response to nutrient levels	4.20E-02	3
GO:1903351	cellular response to dopamine	4.20E-02	5
GO:1903350	response to dopamine	4.38E-02	5
GO:0051047	positive regulation of secretion	4.51E-02	10
GO:0106027	neuron projection organization	4.51E-02	5
GO:0002042	cell migration involved in sprouting angiogenesis	4.51E-02	4
GO:0007389	pattern specification process	4.63E-02	12
GO:0022029	telencephalon cell migration	4.76E-02	4
GO:0010976	positive regulation of neuron projection development	4.89E-02	9
GO:0048520	positive regulation of behavior	4.91E-02	3
GO:0007586	digestion	4.96E-02	6
GO:0097060	synaptic membrane	1.38E-08	22
GO:0045211	postsynaptic membrane	1.38E-08	19

GO:0099572	postsynaptic specialization	5.65E-07	19
GO:0099055	integral component of postsynaptic membrane	2.01E-06	11
GO:0098978	glutamatergic synapse	2.01E-06	18
GO:0098984	neuron to neuron synapse	2.01E-06	18
GO:0098936	intrinsic component of postsynaptic membrane	2.01E-06	11
GO:0099699	integral component of synaptic membrane	2.01E-06	12
GO:0034702	ion channel complex	2.01E-06	16
GO:0014069	postsynaptic density	2.01E-06	17
GO:0032279	asymmetric synapse	2.37E-06	17
GO:0099240	intrinsic component of synaptic membrane	3.70E-06	12
GO:1902495	transmembrane transporter complex	4.24E-06	16
GO:1990351	transporter complex	5.16E-06	16
GO:0008076	voltage-gated potassium channel complex	5.16E-06	9
GO:0099634	postsynaptic specialization membrane	6.96E-06	10
GO:0034703	cation channel complex	7.01E-06	13
GO:0034705	potassium channel complex	9.17E-06	9
GO:0099060	integral component of postsynaptic specialization membrane	1.34E-05	8
GO:0098948	intrinsic component of postsynaptic specialization membrane	1.73E-05	8
GO:0098839	postsynaptic density membrane	6.37E-05	8
GO:0042734	presynaptic membrane	6.46E-05	10
GO:0099061	integral component of postsynaptic density membrane	1.58E-04	6
GO:0099146	intrinsic component of postsynaptic density membrane	2.12E-04	6
GO:0032590	dendrite membrane	6.47E-04	5
GO:0099056	integral component of presynaptic membrane	1.18E-03	6
GO:0098889	intrinsic component of presynaptic membrane	2.14E-03	6
GO:0032589	neuron projection membrane	3.31E-03	5
GO:0098845	postsynaptic endosome	3.97E-03	3
GO:0043197	dendritic spine	4.26E-03	8
GO:0044309	neuron spine	4.45E-03	8
GO:0031902	late endosome membrane	4.63E-03	7
GO:0098563	intrinsic component of synaptic vesicle membrane	1.00E-02	4
GO:0060076	excitatory synapse	1.54E-02	4
GO:0031256	leading edge membrane	1.58E-02	7
GO:0030140	trans-Golgi network transport vesicle	2.80E-02	3
GO:0030285	integral component of synaptic vesicle membrane	3.25E-02	3
GO:0044304	main axon	3.45E-02	4
GO:0043204	perikaryon	3.70E-02	6
GO:0005770	late endosome	4.62E-02	8
GO:0005184	neuropeptide hormone activity	2.22E-06	7
GO:0005249	voltage-gated potassium channel activity	2.22E-06	10
GO:0022843	voltage-gated cation channel activity	2.22E-06	12
GO:0005267	potassium channel activity	3.18E-06	11
GO:0022836	gated channel activity	4.23E-06	17
GO:0005244	voltage-gated ion channel activity	5.18E-06	13
GO:0022832	voltage-gated channel activity	5.18E-06	13
GO:0005216	ion channel activity	1.50E-05	18
GO:0015079	potassium ion transmembrane transporter activity	2.06E-05	11
GO:0015267	channel activity	4.79E-05	18
GO:0022803	passive transmembrane transporter activity	4.79E-05	18
GO:0005179	hormone activity	1.05E-04	9
GO:0046873	metal ion transmembrane transporter activity	1.76E-04	16
GO:0005261	cation channel activity	1.76E-04	14
GO:0015077	monovalent inorganic cation transmembrane transporter activity	2.20E-03	13
GO:0035254	glutamate receptor binding	2.20E-03	5
GO:0008227	G protein-coupled amine receptor activity	2.51E-03	5
GO:0030594	neurotransmitter receptor activity	2.51E-03	7
GO:0030545	receptor regulator activity	2.51E-03	15
GO:0004683	calmodulin-dependent protein kinase activity	3.37E-03	4
GO:0005516	calmodulin binding	3.66E-03	9
GO:0048018	receptor ligand activity	3.71E-03	14
GO:0030546	signaling receptor activator activity	3.98E-03	14
GO:0008195	phosphatidate phosphatase activity	4.24E-03	3
GO:0004993	G protein-coupled serotonin receptor activity	4.24E-03	4
GO:0005251	delayed rectifier potassium channel activity	4.24E-03	4
GO:0099589	serotonin receptor activity	4.24E-03	4

GO:0042577	lipid phosphatase activity	4.70E-03	3
GO:0099095	ligand-gated anion channel activity	1.16E-02	3
GO:0051428	peptide hormone receptor binding	1.52E-02	3
GO:0001965	G-protein alpha-subunit binding	2.77E-02	3
GO:0016247	channel regulator activity	4.18E-02	6
GO:0015108	chloride transmembrane transporter activity	4.18E-02	5

Supplementary Table 8 GO enrichment analysis of differentially upregulated genes in Network D (anterior cingulate network).

ID	Description	BH	Count
GO:0007611	learning or memory	9.24E-06	19
GO:0006813	potassium ion transport	2.46E-05	17
GO:0050890	cognition	2.46E-05	19
GO:0021895	cerebral cortex neuron differentiation	2.46E-05	7
GO:0007187	G protein-coupled receptor signaling pathway, coupled to cyclic nucleotide second messenger	3.25E-05	17
GO:0050804	modulation of chemical synaptic transmission	3.25E-05	23
GO:0099177	regulation of trans-synaptic signaling	3.25E-05	23
GO:0008306	associative learning	3.27E-05	10
GO:0021877	forebrain neuron fate commitment	3.65E-05	5
GO:0021872	forebrain generation of neurons	3.65E-05	9
GO:0034765	regulation of ion transmembrane transport	4.02E-05	23
GO:0071805	potassium ion transmembrane transport	4.19E-05	15
GO:0021892	cerebral cortex GABAergic interneuron differentiation	4.59E-05	5
GO:0021879	forebrain neuron differentiation	7.92E-05	8
GO:0030900	forebrain development	9.39E-05	20
GO:0050806	positive regulation of synaptic transmission	1.34E-04	13
GO:0007612	learning	1.34E-04	12
GO:0097154	GABAergic neuron differentiation	1.34E-04	5
GO:0099601	regulation of neurotransmitter receptor activity	1.34E-04	9
GO:0001662	behavioral fear response	1.34E-04	7
GO:0002209	behavioral defense response	1.52E-04	7
GO:0042596	fear response	1.73E-04	7
GO:0010469	regulation of signaling receptor activity	2.11E-04	12
GO:0021953	central nervous system neuron differentiation	2.66E-04	13
GO:0048167	regulation of synaptic plasticity	2.71E-04	13
GO:0021543	pallium development	5.20E-04	12
GO:1903524	positive regulation of blood circulation	7.05E-04	8
GO:0043266	regulation of potassium ion transport	8.09E-04	9
GO:0007188	adenylate cyclase-modulating G protein-coupled receptor signaling pathway	1.35E-03	13
GO:0021537	telencephalon development	1.38E-03	14
GO:1904062	regulation of cation transmembrane transport	1.46E-03	16
GO:0010959	regulation of metal ion transport	1.67E-03	17
GO:0007193	adenylate cyclase-inhibiting G protein-coupled receptor signaling pathway	1.67E-03	8
GO:0045165	cell fate commitment	1.79E-03	14
GO:2001257	regulation of cation channel activity	2.56E-03	11
GO:0048663	neuron fate commitment	2.74E-03	7
GO:0031644	regulation of nervous system process	2.81E-03	10
GO:0007613	memory	3.31E-03	9
GO:0019932	second-messenger-mediated signaling	3.57E-03	18
GO:0032412	regulation of ion transmembrane transporter activity	3.68E-03	13
GO:0021542	dentate gyrus development	3.95E-03	4
GO:0015837	amine transport	4.37E-03	8
GO:0098664	G protein-coupled serotonin receptor signaling pathway	4.77E-03	5
GO:0022898	regulation of transmembrane transporter activity	5.17E-03	13
GO:0045907	positive regulation of vasoconstriction	5.31E-03	5
GO:0033555	multicellular organismal response to stress	5.35E-03	7
GO:0007218	neuropeptide signaling pathway	5.84E-03	8
GO:0007586	digestion	5.84E-03	9
GO:0032409	regulation of transporter activity	6.96E-03	13
GO:0021761	limbic system development	6.96E-03	8
GO:0007210	serotonin receptor signaling pathway	6.96E-03	5

GO:0021954	central nervous system neuron development	6.96E-03	7
GO:1901379	regulation of potassium ion transmembrane transport	6.96E-03	7
GO:0009914	hormone transport	7.20E-03	14
GO:0021885	forebrain cell migration	7.56E-03	6
GO:0051260	protein homooligomerization	8.47E-03	10
GO:0021987	cerebral cortex development	1.02E-02	8
GO:0060041	retina development in camera-type eye	1.02E-02	9
GO:1900449	regulation of glutamate receptor signaling pathway	1.02E-02	6
GO:0051952	regulation of amine transport	1.27E-02	7
GO:0031646	positive regulation of nervous system process	1.28E-02	6
GO:0003002	regionalization	1.54E-02	14
GO:0035249	synaptic transmission, glutamatergic	1.57E-02	7
GO:0045600	positive regulation of fat cell differentiation	1.57E-02	6
GO:0010460	positive regulation of heart rate	1.83E-02	4
GO:0007215	glutamate receptor signaling pathway	1.94E-02	7
GO:0007389	pattern specification process	2.00E-02	16
GO:0009582	detection of abiotic stimulus	2.34E-02	8
GO:0045666	positive regulation of neuron differentiation	2.43E-02	14
GO:0016322	neuron remodeling	2.65E-02	3
GO:0035296	regulation of tube diameter	2.65E-02	8
GO:0097746	regulation of blood vessel diameter	2.65E-02	8
GO:1903522	regulation of blood circulation	2.65E-02	12
GO:0042310	vasoconstriction	2.65E-02	6
GO:0035150	regulation of tube size	2.70E-02	8
GO:0021772	olfactory bulb development	3.13E-02	4
GO:0019935	cyclic-nucleotide-mediated signaling	3.25E-02	10
GO:0021766	hippocampus development	3.28E-02	6
GO:0022029	telencephalon cell migration	3.46E-02	5
GO:0097756	negative regulation of blood vessel diameter	3.55E-02	6
GO:0046883	regulation of hormone secretion	3.55E-02	11
GO:0003018	vascular process in circulatory system	3.55E-02	9
GO:0008217	regulation of blood pressure	3.55E-02	9
GO:0060347	heart trabecula formation	3.55E-02	3
GO:0021988	olfactory lobe development	3.56E-02	4
GO:0007409	axonogenesis	3.65E-02	16
GO:1901016	regulation of potassium ion transmembrane transporter activity	3.70E-02	5
GO:0009416	response to light stimulus	3.91E-02	12
GO:0046879	hormone secretion	3.91E-02	12
GO:0048521	negative regulation of behavior	3.96E-02	3
GO:0051259	protein complex oligomerization	3.96E-02	10
GO:0090257	regulation of muscle system process	3.96E-02	10
GO:1903351	cellular response to dopamine	4.00E-02	6
GO:0019229	regulation of vasoconstriction	4.00E-02	5
GO:1903350	response to dopamine	4.15E-02	6
GO:0001505	regulation of neurotransmitter levels	4.33E-02	10
GO:0098962	regulation of postsynaptic neurotransmitter receptor activity	4.50E-02	3
GO:0050803	regulation of synapse structure or activity	4.97E-02	10
GO:0045211	postsynaptic membrane	5.33E-04	16
GO:0008076	voltage-gated potassium channel complex	5.33E-04	9
GO:0034705	potassium channel complex	7.11E-04	9
GO:0097060	synaptic membrane	7.11E-04	18
GO:0043204	perikaryon	4.04E-03	10
GO:0098984	neuron to neuron synapse	9.97E-03	15
GO:0034703	cation channel complex	9.97E-03	11
GO:0034702	ion channel complex	9.97E-03	13
GO:0042734	presynaptic membrane	1.18E-02	9
GO:0099572	postsynaptic specialization	1.47E-02	14
GO:1902495	transmembrane transporter complex	1.47E-02	13
GO:0098978	glutamatergic synapse	1.47E-02	14
GO:1990351	transporter complex	1.58E-02	13
GO:0014069	postsynaptic density	1.89E-02	13
GO:0032279	asymmetric synapse	2.08E-02	13
GO:0099055	integral component of postsynaptic membrane	2.39E-02	7
GO:0099699	integral component of synaptic membrane	2.59E-02	8
GO:0098936	intrinsic component of postsynaptic membrane	2.70E-02	7

GO:0099240	intrinsic component of synaptic membrane	3.63E-02	8
GO:0032590	dendrite membrane	3.63E-02	4
GO:0005249	voltage-gated potassium channel activity	1.12E-04	10
GO:0005267	potassium channel activity	1.40E-04	11
GO:0005184	neuropeptide hormone activity	3.08E-04	6
GO:0022843	voltage-gated cation channel activity	3.08E-04	11
GO:0005516	calmodulin binding	3.08E-04	13
GO:0015079	potassium ion transmembrane transporter activity	6.10E-04	11
GO:0008227	G protein-coupled amine receptor activity	2.54E-03	6
GO:0004993	G protein-coupled serotonin receptor activity	2.54E-03	5
GO:0099589	serotonin receptor activity	2.54E-03	5
GO:0005244	voltage-gated ion channel activity	2.54E-03	11
GO:0022832	voltage-gated channel activity	2.54E-03	11
GO:0015267	channel activity	2.54E-03	18
GO:0022803	passive transmembrane transporter activity	2.54E-03	18
GO:0022836	gated channel activity	6.17E-03	14
GO:0005179	hormone activity	6.17E-03	8
GO:0005216	ion channel activity	6.17E-03	16
GO:0043176	amine binding	1.19E-02	3
GO:0051378	serotonin binding	1.19E-02	3
GO:0001228	DNA-binding transcription activator activity, RNA polymerase II-specific	1.19E-02	17
GO:0004683	calmodulin-dependent protein kinase activity	1.19E-02	4
GO:0001216	DNA-binding transcription activator activity	1.21E-02	17
GO:0071855	neuropeptide receptor binding	1.76E-02	4
GO:0042562	hormone binding	1.76E-02	6
GO:0005261	cation channel activity	3.25E-02	12
GO:0004497	monooxygenase activity	3.25E-02	6
GO:0046873	metal ion transmembrane transporter activity	3.25E-02	14
GO:0051428	peptide hormone receptor binding	4.56E-02	3

Supplementary Table 9 Region-specific acronyms used as column names in heatmaps.

Acronym	Name	Acronym	Name
AOrG	Anterior orbital gyrus	SMG-s	Supramarginal gyrus, superior bank of gyrus
fro	Frontal operculum	SMG-i	Supramarginal gyrus, inferior bank of gyrus
FP-s	Frontal pole, superior aspect	PCLp-cs	Paracentral lobule, posterior part, bank of cingulate sulcus
FPi	Frontal pole, inferior aspect	PoG-cs	Postcentral gyrus, bank of the central sulcus
GRe	Gyrus rectus	PoG-sl	Postcentral gyrus, superior lateral aspect of gyrus
opIFG	Inferior frontal gyrus, opercular part	PoG-il	Postcentral gyrus, inferior lateral aspect of gyrus
orIFG	Inferior frontal gyrus, orbital part	PoG-pcs	Postcentral gyrus, bank of the posterior central sulcus
trIFG	Inferior frontal gyrus, triangular part	Pcu-s	Precuneus, superior lateral bank of gyrus
IRoG	Inferior rostral gyrus	Pcu-i	Precuneus, inferior lateral bank of gyrus
LOrG	Lateral orbital gyrus	FuG-its	Fusiform gyrus, bank of the its
MOrG	Medial orbital gyrus	FuG-l	Fusiform gyrus, lateral bank of gyrus
MFG-s	Middle frontal gyrus, superior bank of gyrus	FuG-cos	Fusiform gyrus, bank of cos
MFG-i	Middle frontal gyrus, inferior bank of gyrus	HG	Heschl's gyrus
PCLa	Paracentral lobule, anterior part	ITG-its	Inferior temporal gyrus, bank of the its
PCLa-s	Paracentral lobule, anterior part, superior bank of gyrus	ITG-l	Inferior temporal gyrus, lateral bank of gyrus
PCLa-i	Paracentral lobule, anterior part, inferior bank of gyrus	ITG-mts	Inferior temporal gyrus, bank of mts
PaOG	Parolfactory gyri	MTG-s	Middle temporal gyrus, superior bank of gyrus
POrG	Posterior orbital gyrus	MTG-i	Middle temporal gyrus, inferior bank of gyrus
PrG-prc	Precentral gyrus, bank of the precentral sulcus	PLP	Planum polare
PrG-sl	Precentral gyrus, superior lateral aspect of gyrus	PLT	Planum temporale
PrG-il	Precentral gyrus, inferior lateral aspect of gyrus	STG-l	Superior temporal gyrus, lateral bank of gyrus
PrG-cs	Precentral gyrus, bank of the central sulcus	STG-i	Superior temporal gyrus, inferior bank of gyrus
SFG-m	Superior frontal gyrus, medial bank of gyrus	TP-s	Temporal pole, superior aspect
SFG-l	Superior frontal gyrus, lateral bank of gyrus	TG	Transverse gyri
SRoG	Superior rostral gyrus	ATZ	Amygdalohippocampal transition zone
LIG	Long insular gyri	BLA	Basolateral nucleus
SIG	Short insular gyri	BMA	Basomedial nucleus
CgGf-s	Cingulate gyrus, frontal part, superior bank of gyrus	CeA	Central nucleus
CgGf-i	Cingulate gyrus, frontal part, inferior bank of gyrus	COMA	Cortico-medial group
CgGp-s	Cingulate gyrus, parietal part, superior bank of gyrus	LA	Lateral nucleus
CgGp-i	Cingulate gyrus, parietal part, inferior bank of gyrus	DBv	Nucleus of the diagonal band, horizontal division
CgGr-s	Cingulate gyrus, retrosplenial part, superior bank of gyrus	OlfT	Olfactory tubercle
CgGr-i	Cingulate gyrus, retrosplenial part, inferior bank of gyrus	GPe	Globus pallidus, external segment
SCG	Subcallosal cingulate gyrus	TCd	Tail of caudate nucleus
DG	Dentate gyrus	Pu	Putamen
CA1	CA1 field	Cl	Claustrum
CA2	CA2 field	SO	Supraoptic nucleus
CA3	CA3 field	Sb	Subthalamic nucleus
CA4	CA4 field	LGd	Dorsal lateral geniculate nucleus
S	Subiculum	DTLv	Lateral group of nuclei, ventral division
PHG-l	Parahippocampal gyrus, lateral bank of gyrus	MG	Medial geniculate complex
PHG-cos	Parahippocampal gyrus, bank of the cos	DTP	Posterior group of nuclei
Pir	Piriform cortex	R	Reticular nucleus of thalamus
Cun-pest	Cuneus, peristriate	SNC	Substantia nigra, pars compacta
IOG-s	Inferior occipital gyrus, superior bank of gyrus	SNR	Substantia nigra, pars reticulata
IOG-i	Inferior occipital gyrus, inferior bank of gyrus	He-Crus I	Crus I, lateral hemisphere
LiG-str	Lingual gyrus, striate	PV-Crus II	Crus II, paravermis
OTG-s	Occipito-temporal gyrus, superior bank of gyrus	He-Crus II	Crus II, lateral hemisphere
OTG-i	Occipito-temporal gyrus, inferior bank of gyrus	PV-VIIB	VIIB, paravermis
SOG-s	Superior occipital gyrus, superior bank of gyrus	He-VIIB	VIIB, lateral hemisphere
SOG-i	Superior occipital gyrus, inferior bank of gyrus	He-VIIIA	VIIIA, lateral hemisphere
AnG-i	Angular gyrus, inferior bank of gyrus	CPLV	Choroid plexus of the lateral ventricle

Supplementary Figures

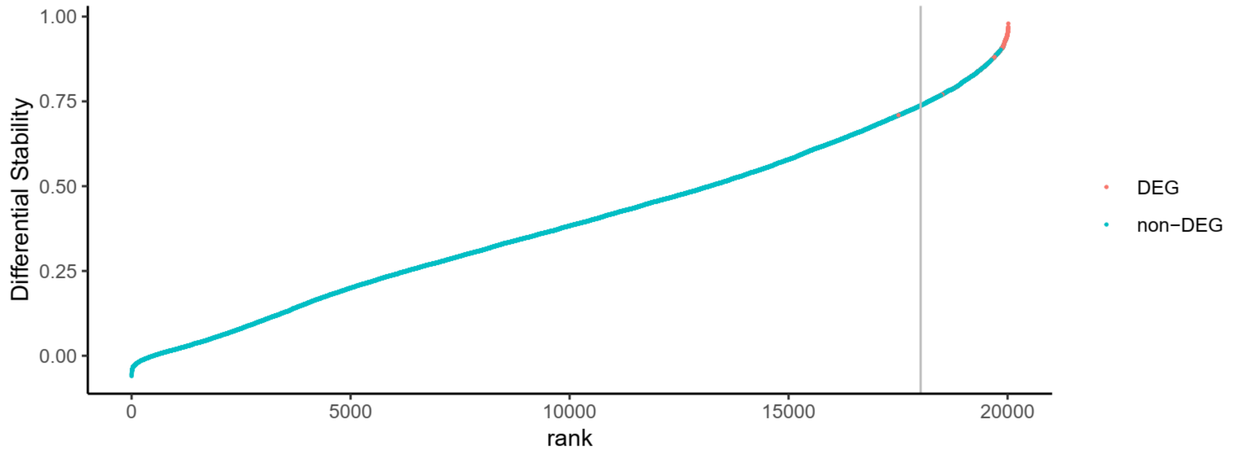

Supplementary Figure 1 Differential stability values for all 20,017 genes. At least 92% of the differentially expressed genes (DEGs) in Network C and Network D were among the top decile of all genes (gray vertical line) and correspond to differential stability values > 0.73.

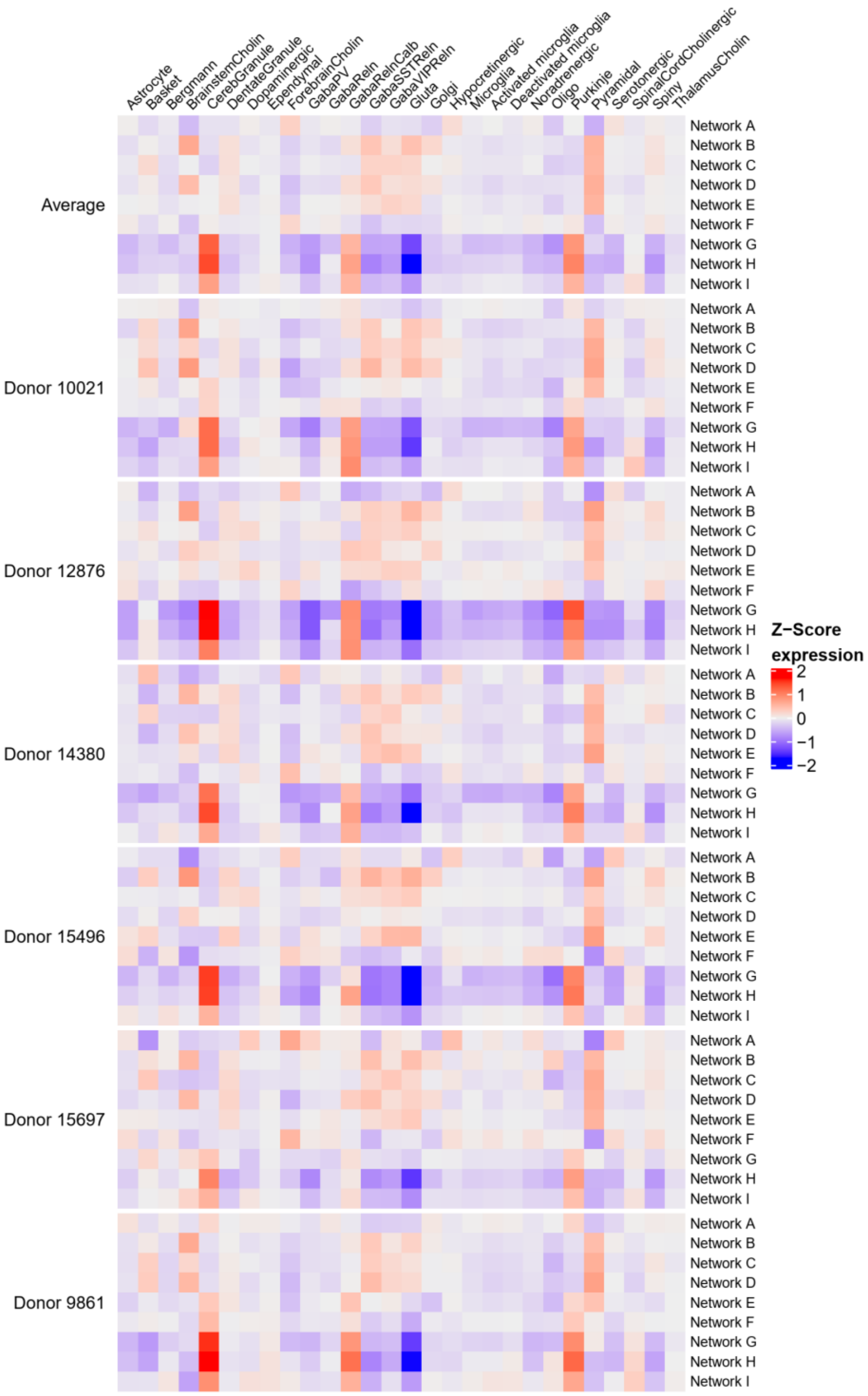

Supplementary Figure 2 Expression of cell-types in anatomical networks. Gene expression was Z-scored and averaged across cell-type specific markers and across samples within anatomical networks. First heatmap shows the element-wise average expression across the six donors in the Allen Human Brain Atlas for which heatmaps are also shown for each donor.

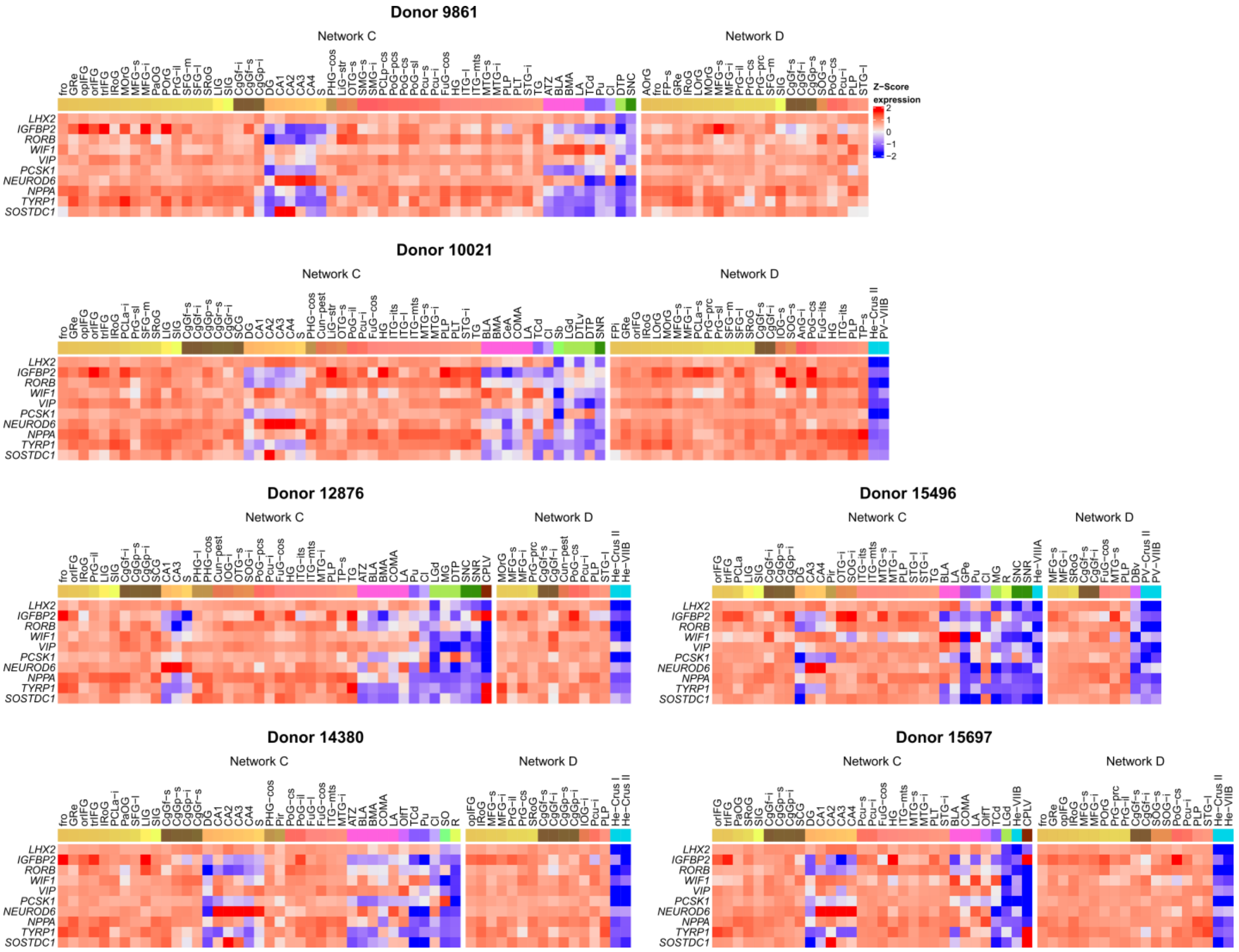

Supplementary Figure 3 Expression of differentially upregulated marker genes in Network C (posterior cingulate network) and Network D (anterior cingulate network). Heatmaps of marker genes (rows) are shown for each one of the six donors in the Allen Human Brain Atlas with color annotation for anatomical structures (columns). Expression was averaged across samples with the same acronym ignoring left and right hemisphere annotations. See Supplementary Table 7 for region-specific acronyms.

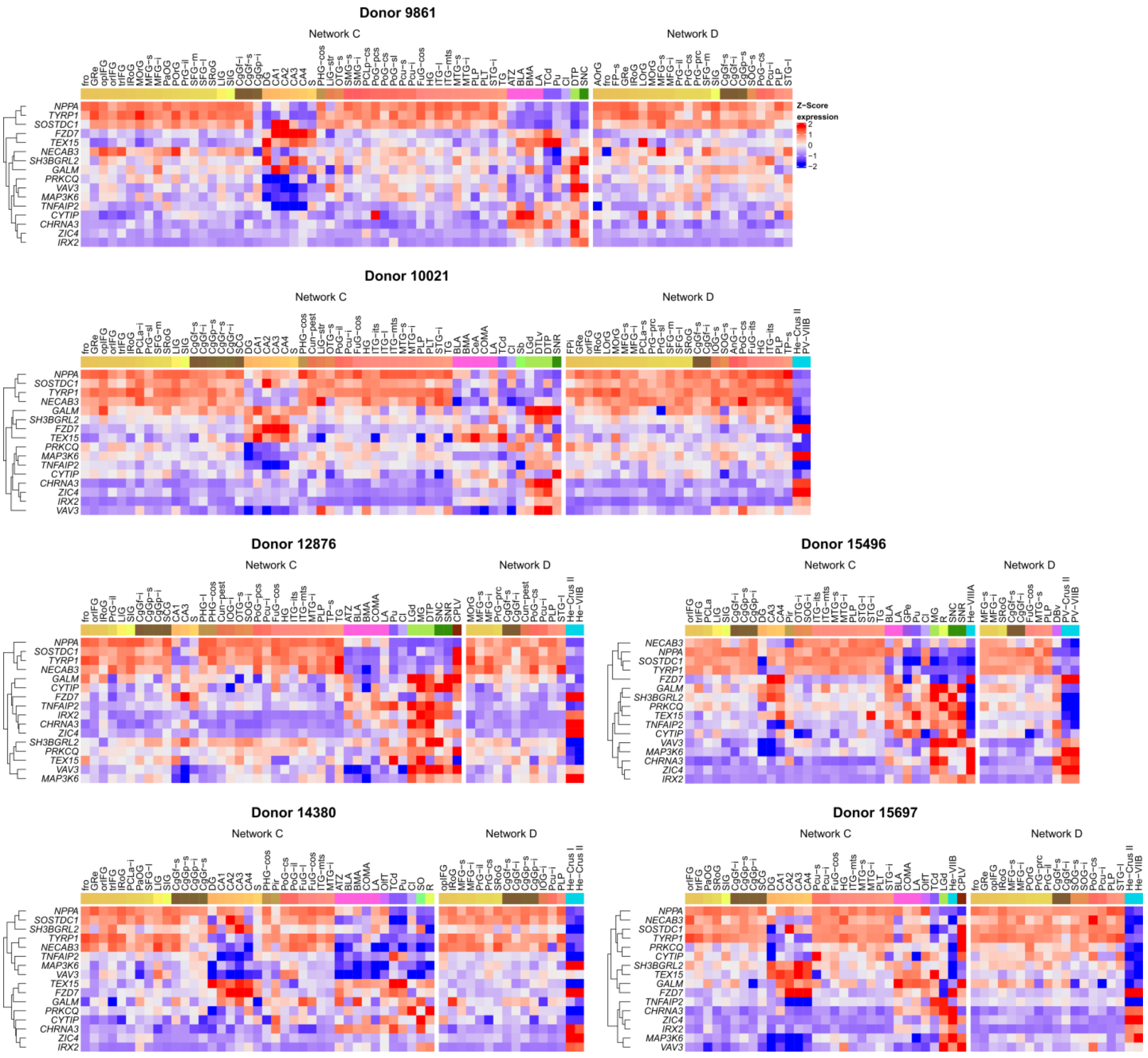

Supplementary Figure 4 Expression of thalamus cholinergic marker genes in Network C (posterior cingulate network) and Network D (anterior cingulate network). Heatmaps are shown for each one of the six donors in the Allen Human Brain Atlas with color annotation for anatomical structures (columns). Expression was averaged across samples with the same acronym ignoring left and right hemisphere annotations. Marker genes (rows) were clustered with complete linkage separately for each network. See Supplementary Table 7 for region-specific acronyms.
